# The most abundant cyst wall proteins of *Acanthamoeba castellanii* are lectins that bind cellulose and localize to distinct structures in developing and mature cyst walls

**DOI:** 10.1371/journal.pntd.0007352

**Published:** 2019-05-16

**Authors:** Pamela Magistrado-Coxen, Yousuf Aqeel, Angelo Lopez, John R. Haserick, Breeanna R. Urbanowicz, Catherine E. Costello, John Samuelson

**Affiliations:** 1 Department of Molecular and Cell Biology, Boston University Goldman School of Dental Medicine, Boston, Massachusetts, United States of America; 2 Department of Biochemistry, Boston University School of Medicine, Boston, Massachusetts, United States of America; 3 Complex Carbohydrate Research Center, University of Georgia, Athens, Georgia, United States of America; Johns Hopkins Bloomberg School of Public Health, UNITED STATES

## Abstract

**Background:**

*Acanthamoeba castellanii*, which causes keratitis and blindness in under-resourced countries, is an emerging pathogen worldwide, because of its association with contact lens use. The wall makes cysts resistant to sterilizing reagents in lens solutions and to antibiotics applied to the eye.

**Methodology/Principal findings:**

Transmission electron microscopy and structured illumination microscopy (SIM) showed purified cyst walls of *A*. *castellanii* retained an outer ectocyst layer, an inner endocyst layer, and conical ostioles that connect them. Mass spectrometry showed candidate cyst wall proteins were dominated by three families of lectins (named here Jonah, Luke, and Leo), which bound well to cellulose and less well to chitin. An abundant Jonah lectin, which has one choice-of-anchor A (CAA) domain, was made early during encystation and localized to the ectocyst layer of cyst walls. An abundant Luke lectin, which has two carbohydrate-binding modules (CBM49), outlined small, flat ostioles in a single-layered primordial wall and localized to the endocyst layer and ostioles of mature walls. An abundant Leo lectin, which has two unique domains with eight Cys residues each (8-Cys), localized to the endocyst layer and ostioles. The Jonah lectin and glycopolymers, to which it binds, were accessible in the ectocyst layer. In contrast, Luke and Leo lectins and the glycopolymers, to which they bind, were mostly inaccessible in the endocyst layer and ostioles.

**Conclusions/Significance:**

The most abundant *A*. *castellanii* cyst wall proteins are three sets of lectins, which have carbohydrate-binding modules that are conserved (CBM49s of Luke), newly characterized (CAA of Jonah), or unique to *Acanthamoebae* (8-Cys of Leo). Cyst wall formation is a tightly choreographed event, in which lectins and glycopolymers combine to form a mature wall with a protected endocyst layer. Because of its accessibility in the ectocyst layer, an abundant Jonah lectin is an excellent diagnostic target.

## Author summary

A half century ago, investigators identified cellulose in the *Acanthamoeba* cyst wall, which has two layers and conical ostioles that connect them. Here we showed cyst walls contain three large sets of cellulose-binding lectins, which localize to the ectocyst layer (a Jonah lectin) or to the endocyst layer and ostioles (Luke and Leo lectins). We used the lectins to establish a sequence for cyst wall assembly when trophozoites are starved and encyst. In the first stage, a Jonah lectin and glycopolymers were present in dozens of distinct vesicles. In the second stage, a primordial wall contained small, flat ostioles outlined by a Luke lectin. In the third stage, a Jonah lectin remained in the ectocyst layer, while Luke and Leo lectins moved to the endocyst layer and ostioles. A description of the major events during cyst wall development is a starting point for mechanistic studies of its assembly.

## Introduction

*Acanthamoebae*, which include the genome project *A*. *castellanii* Neff strain, are soil protists named for acanthopods (spikes) on the surface of trophozoites [1]. In immunocompetent persons, *Acanthamoeba* is a rare but important cause of corneal inflammation (keratitis), which is difficult to treat and so may lead to scarring and blindness [2–4]. In immunosuppressed patients, *Acanthamoeba* may cause encephalitis [5]. *Acanthamoeba* is endemic in under-resourced populations in the Middle East, South Asia, Africa, and Latin America [6–11]. *Acanthamoeba* is an emerging pathogen in Europe, North America, and Australia, where 80 to 90% of infections are associated with contact lens use [12–14]. Because water for washing hands may be scarce in places where the free-living protist is frequent, we recently showed that alcohols in concentrations present in hand sanitizers kill *A*. *castellanii* trophozoites and cysts [15, 16].

When *A*. *castellanii* trophozoites are deprived of nutrients in solution or on agar plates, they form cysts [17–19]. Transmission electron microscopy (TEM) shows cyst walls have two microfibril-dense layers (outer ectocyst and inner endocyst), which are separated by a relatively microfibril-free layer [20]. The endocyst and ectocyst layers are connected to each other by conical ostioles, through which the protist escapes during excystation [21].

The cyst wall of *A*. *castellanii* protects free-living protists from osmotic shock when exposed to fresh water, drying when exposed to air, or starvation when deprived of bacteria or other food sources. The cyst wall also acts as a barrier, sheltering parasites from killing by disinfectants used to clean surfaces, sterilizing agents in contact lens solutions, and/or antibiotics applied directly to the eye [22–24].

We are interested in the cyst wall proteins of *A*. *castellanii* for three reasons. First, although monoclonal antibodies to *A*. *castellanii* have been made, the majority react to trophozoites, and no cyst wall proteins have been molecularly identified [25–27]. Indeed the only cyst-specific protein identified, which was named for its 21-kDa predicted size (CSP21), is unlikely to be a cyst wall protein, as it lacks a signal peptide [28]. A cyst wall protein that is unique, abundant, accessible, and conserved across many strains of *Acanthamoebae* would therefore be an excellent target for a new diagnostic antibody. Second, *A*. *castellanii* and related species are the only human pathogens that contain cellulose in their wall [29–31]. *Dictyostelium discoideum*, which also has cellulose in its walls, is not a significant pathogen [32]. In addition, the whole genome of *A*. *castellanii* predicts a set of candidate cyst wall proteins that contain two or three carbohydrate-binding modules (CBM49s), which are homologs of a C-terminal cellulose-binding domain (SlCBM49) of the *Solanum lycopersicum* (tomato) endocellulase SlGH9C [1, 33–36]. Further, the genome predicts a chitin (a polymer of β-1,4-linked GlcNAc) synthase, a chitinase, and two chitin deacetylases, suggesting the possibility that chitin and chitin-binding proteins are also present in the cyst wall [37]. Note, however, that monosaccharide analysis of cyst wall glycopolymers revealed β-1,4-linked glucose and galactose rather than GlcNAc [31]. Third, we are interested in whether abundant cyst wall proteins localize to particular structures in the mature wall: ectocyst layer, endocyst layer, and ostioles. If so, the location of these proteins at numerous time points during encystation might provide insights into how the cyst wall is assembled.

Our experimental design was relatively simple. We used TEM, as well as structured illumination microscopy (SIM) and probes for glycopolymers, to judge the intactness and cleanliness of purified *A*. *castellanii* cyst walls [38]. We used mass spectrometry to identify candidate cyst wall proteins, which were compared to proteins present in walls of other protists, bacteria, fungi, and plants [39]. We used SIM to localize abundant cyst wall proteins, each of which was tagged with a green fluorescent protein (GFP) and expressed under its own promoter, in encysting protists and in mature cysts [40, 41]. We also determined whether each cyst wall protein, expressed as a GFP-tagged protein under a constitutive glyceraldehyde 3-phosphate dehydrogenase (GAPDH) promoter in trophozoites or as a maltose-binding protein (MBP) in the periplasm of *Escherichia coli*, binds to microcrystalline cellulose and/or chitin beads [42]. Finally, we used anti-GFP antibodies and MBP-cyst wall protein fusions to test the accessibility of proteins and glycopolymers, respectively, in the ectocyst and endocyst layers of mature walls.

In this way, we began to answer five basic questions concerning *A*. *castellanii* cyst wall proteins: What are their identities? When are they made? Where are cyst wall proteins located in the developing and mature cyst wall? Why are they located there? Which cyst wall protein is the best target for diagnostic antibodies?

## Methods

### Ethics statement

Culture and manipulation of *A*. *castellanii* were approved by the Boston University Institutional Biosafety Committee.

### Culture of trophozoites and preparation of encysting organisms and cysts

*A*. *castellanii* Neff strain trophozoites were purchased from the American Type Culture collection. Trophozoites of *A*. *castellanii* MEEI 0184 strain, which was derived from a human corneal infection, were obtained from Dr. Noorjahan Panjwani of Tufts University School of Medicine [16]. Neff strain organisms were used for all experiments with the exception of a few initial mass spectrometric studies. Trophozoites were grown in T-75 tissue culture flasks at 30°C in 10 ml ATCC medium 712 (PYG plus additives) (Sigma-Aldrich Corporation, St. Louis, MO) [18]. Flasks containing log-phase trophozoites (free of cysts that form spontaneously in stationary cultures) were either chilled or scraped with a cell scraper to release adherent amoebae, which were concentrated by centrifugation at 500 x g for 5 min and washed twice with phosphate buffered saline (PBS). Approximately 10^7^ amoebae obtained from a confluent flask were induced to encyst by incubation at 30°C on agar plates containing non-nutrient medium, which contained 2% agarose [16]. After 3, 6, 12, 15, 18, 24, 36, 72, or 144 hr incubation, 15 ml of PBS was added to agar plates, which were incubated on a shaker for 30 min at room temperature (RT). Encysting organisms were removed using a cell scraper and concentrated by centrifugation for 10 min at 500 x g for <24 hr cysts or at 1,500 x g for >24 hr cysts. Nearly 100% of the organisms formed cysts.

### Preparation of mature cyst walls for SIM, TEM, and mass spectrometry

Between 5 and 10 million mature cysts (after 144 hr encystation) were washed in PBS and suspended in lysis buffer (10 mM HEPES, 25 mM KCl, 1 mM CaCl_2_, 10 mM MgCl_2_, 2% CHAPS, and 1X Roche protease inhibitor) (Sigma-Aldrich). For SIM, cysts in 500-μl lysis buffer were broken four times for 2 min each with 200 μl of 0.5 mm glass beads in a Mini-Beadbeater-16 (BioSpec Products, Bartlesville, OK). For TEM, where glass beads cannot be used, cysts in 200-μl lysis buffer were broken by sonication four times for 20 seconds each in continuous mode in a Sonicator Cell Disruptor (formerly Heat Systems Ultrasonic, now Qsonica, Newtown, CT). Broken cysts were added to the top a 15-ml falcon tube containing 60% sucrose in _dd_H_2_O and centrifuged at 4,000 x g for 10 min. Bead beating breaks 95 to 100% of cysts. The broken cyst wall pellet, which contained zero to 5% cysts, was suspended in PBS buffer and washed three times at 10,000 x g in a microcentrifuge. The cyst wall pellet was used without further modification for SIM or TEM.

For mass spectrometry, the cyst wall pellet was placed at the top of gradient containing 2 ml each of 20%, 40%, 60% and 80% Percoll (top to bottom), which was buffered with PBS, and centrifuged for 20 min at 3,000 x g. The layer between 60% and 80% Percoll, where the broken cyst walls were located, was collected and washed in PBS. The cyst wall preparation was suspended in 10 ml of PBS, placed in a syringe, and forced through a 25-mm diameter Whatman Nuclepore Track-Etched Membrane with 8-μm holes (Sigma-Aldrich). The cellular debris, which passed through the membranes, was discarded. The membrane was removed from the cassette, suspended in 5 ml of PBS, and vortexed to release cyst walls. The membrane was removed, and cyst walls were distributed in microfuge tubes and pelleted at 15,000 x g for 10 min. The pellet was suspended in 50 μl PBS and stored at -20°C prior to trypsin digestion and mass spectrometry analysis.

### SIM of glycopolymers of mature cysts and purified cyst walls

A GST-AcCBM49 fusion-construct, which contains the N-terminal CBM49 of an abundant Luke(2) lectin minus the signal peptide, was prepared by codon optimization (76 to 330-bp coding region of ACA1_377670) (S1 Fig and S1 Excel file) (GenScript, Piscataway, NJ). It was cloned into pGEX-6p-1 (GE Healthcare Life Sciences, Marlborough, MA) for cytoplasmic expression in BL21(DE3) chemically competent *E*. *coli* (Thermo Fisher Scientific, Waltham, MA) [43]. Expression of GST-AcCBM49 and GST were induced with 1 mM IPTG for 4 hr at RT, and GST-fusions were purified on glutathione-agarose and conjugated to Alexa Fluor 594 succinimidyl esters (red) (Molecular Probes, Thermo Fisher Scientific). Approximately 10^6^ mature cysts or cyst walls were washed in PBS and fixed in 1% paraformaldehyde buffered with 0.2 M phosphate, pH 7.5, for 15 min at RT. Pellets were washed two times with Hank’s Buffered Saline Solution (HBSS) and incubated with HBSS containing 1% bovine serum albumin (BSA) for 1 hour at RT. Preparations were then incubated for 2 hr at 4°C with 10 μl of 0.25 μg/μl GST-CBM49 conjugated to Alexa Fluor 594 and 20 μl of 0.625 μg/μl wheat germ agglutinin (WGA) (Vector Laboratories, Burlingame, CA) conjugated to Alexa Fluor 488 in 100 μl HBSS [44, 45]. Finally, pellets were labeled with 100 μg of calcofluor white M2R (CFW) (Sigma-Aldrich) in 100 μl HBSS for 15 min at RT and washed five times with HBSS [46, 47]. Preparations were mounted in Mowiol mounting medium (Sigma-Aldrich) and observed with widefield and differential interference contrast microscopy, using a 100x objective of a Zeiss AXIO inverted microscope with a Colibri LED (Carl Zeiss Microcopy LLC, Thornwood, NY). Images were collected at 0.2-μm optical sections with a Hamamatsu Orca-R2 camera and deconvolved using ZEN software (Zeiss). Alternatively, SIM was performed with a 63-x objective of a Zeiss ELYRA S.1 microscope at Boston College (Chestnut Hill, MA), and 0.09-μm optical sections deconvolved using Zen software [38]. All SIM images shown were 3D reconstructions using dozens of z-stacks.

### TEM of mature cysts and purified walls

High-pressure freezing and freeze substitution were used to prepare cysts and cyst walls for TEM at the Harvard Medical School Electron Microscope facility [48]. To make them noninfectious, we fixed mature cysts in 1% paraformaldehyde in PBS for 10 min at RT and washed them two times in PBS. Cyst walls in PBS were pelleted, placed in 6-mm Cu/Au carriers, and frozen in an EM ICE high-pressure freezer (Leica Microsystems, Buffalo Grove, Il). Freeze substitution was performed in a Leica EM AFS2 instrument in dry acetone containing 1% _dd_H_2_0, 1% OsO_4_, and 1% glutaraldehyde at -90°C for 48 hr. The temperature was increased 5°C/hour to 20°C, and samples were washed 3 times in pure acetone and once in propylene oxide for 10 min each. Samples were infiltrated with 1:1 Epon:propylene oxide overnight at 4°C and embedded in TAAB Epon (Marivac Canada Inc. St. Laurent, Canada). Ultrathin sections (80 to 100 nm thick) were cut on a Leica Reichert Ultracut S microtome, picked up onto copper grids, stained with lead citrate, and examined in a JEOL 1200EX transmission electron microscope (JEOL USA, Peabody, MA). Images were recorded with an AMT 2k CCD camera.

### Liquid chromatography-mass spectrometry (LC-MS/MS) of tryptic and chymotryptic peptides from cyst walls

Approximately 10 million broken cyst walls, prepared as above, were dissolved into 50 mM NH_4_HCO_3_, pH 8.0, reduced with 10 mM dithiothreithol (DTT) for 20 min at 60°C, alkylated with 55 mM iodoacetamide (IAA) for 20 min at RT, and then digested with proteomics grade trypsin (Sigma-Aldrich) overnight at 37°C. Alternatively broken cyst walls either before or after digestion with trypsin were reconstituted in 1× reducing SDS/PAGE loading buffer and run on a 4–20% precast polyacrylamide TGX gel (Bio-Rad Laboratories, Philadelphia, PA). Bands stained by colloidal Coomassie blue were excised and washed with 50 mM NH_4_HCO_3_/acetonitrile (ACN). Reduction, alkylation, and trypsin/chymotrypsin digestion were performed in-gel. Peptides were dried and desalted using C18 ZipTip concentrators (MilliporeSigma, Burlington, MA). Peptides from five biological replicates for both *in solution* and *in-situ* hydrolyses were dissolved in 2% ACN, 0.1% formic acid (FA) and separated using a nanoAcquity-UPLC system (Waters Corporation, Milford, MA) equipped with a 5-μm Symmetry C18 trap column (180 μm x 20 mm) and a 1.7-μm BEH130 C18 analytical column (150 μm × 100 mm). Samples were loaded onto the precolumn and washed for 4 min at a flow rate of 4 μl/min with 100% mobile phase A (99% water/1% ACN/0.1% FA). Samples were eluted to the analytical column with a gradient of 2–40% mobile phase B (99% ACN/1% water/0.1% FA) delivered over 40 or 90 min at a flow rate of 0.5 μl/min. The analytical column was connected online to a QE or a QE-HF Mass Spectrometer (Thermo Fisher Scientific) equipped with a Triversa NanoMate (Advion Inc., Ithaca, NY) electrospray ionization (ESI) source, which was operated at 1.7 kV. Data were acquired in automatic Data Dependent top 10 (QE) or top 20 (QE-HF) mode. Automated database searches were performed using the PEAKS software suite version 8.5 (Bioinformatics Solutions Inc., Waterloo, ON, Canada). The predicted proteins of *Acanthamoeba castellanii* Neff strain (AmoebaDB-33June 30, 2017) was used to predict tryptic peptides for mass spectrometric analyses and was used for bioinformatics analyses (see below) [36]. The search criteria were set as follows: trypsin/chymotrypsin as the enzyme with ≤ 3 missed cleavages and ≤ 1 non-specific cleavage, the error tolerances for the precursor of 5 ppm and 0.05 Da for fragment ions, carbamidomethyl cysteine as a fixed modification, oxidation of methionine, Pyro-glu from glutamine, and deamidation of asparagine or glutamine as variable modifications. The peptide match threshold (-10 logP) was set to 15, and only proteins with a minimum of two unique peptides were considered. The mass spectrometry proteomics data have been deposited to the ProteomeXchange Consortium (http://proteomecentral.proteomexchange.org) via the PRIDE partner repository with the dataset identifier PXD011826 [49].

### Bioinformatic characterization of candidate cyst wall proteins

Signal peptides and transmembrane helices were predicted using SignalP 4.1 and TMHMM, respectively [50, 51]. Glycosylphosphatidylinositol anchors were searched using big-PI [52]. AmoebaDB, which contains sequence information from the Neff strain and ten other *Acanthamoeba* strains, was used to identify genome sequences, predict introns, and identify paralogous proteins [35, 36]. The NR database at the NCBI was used to identify homologs of candidate cyst wall proteins in other species and to identify conserved domains [53]. Carbohydrate-binding modules were searched using CAZy and InterPro databases [34, 54, 55].

### Expression and visualization of GFP-fusions in transfected *A*. *castellanii*

We used RT-PCR from RNA of encysting protists to obtain the coding sequences of an abundant Luke(2) lectin (840-bp CDS of ACA1_377670), Leo lectin (562-bp CDS of ACA1_074730), and Jonah(1) lectin (1596-bp CDS of ACA1_164810). An abundant Luke(3) lectin (1293-bp CDS of ACA1_245650) did not contain any introns and so was obtained from genomic DNA. Please see S1 Excel file for a list of primers used to make all the constructs. Using NEBuilder HiFi DNA assembly (New England Biolabs, Ipswich, MA), we cloned each CDS into the pGAPDH plasmid, which was a kind gift from Yeonchul Hong of Kyongpook National University School of Medicine, Deagu, Korea [41]. pGAPDH contains a neomycin resistance gene under a TATA-box promoter (for selection with G418) and a glyceraldehyde 3-phosphate dehydrogenase promoter for constitutive expression of GFP-fusions (S1 Fig). The GFP tag was placed at the C-terminus of each cyst wall protein, and a polyadenylation sequence was added downstream of the GFP-fusion’s stop codon. For expression of cyst wall protein genes under their own promoters, we replaced the GAPDH promoter with 446-bp from the 5 ‘UTR of the Luke(2) gene, 500-bp from the 5’ UTR of the Luke(3) gene, 486-bp from the 5’ UTR of the Leo gene, and 571-bp of the 5’UTR of the Jonah(1) gene, each cloned from the genomic DNA. As controls, GFP alone and SP-GFP, which contains a 60-bp sequence encoding an N-terminal signal peptide of Luke(2) lectin, were expressed under a GAPDH promoter. As another control, the 470-bp 5’ UTR and 525-bp CDS of CSP21 (ACA1_075240) was made with a GFP tag [28].

Transfections in *A*. *castellanii* were performed as described previously [40, 41] with some modifications. Briefly, 5 x 10^5^ log-phase trophozoites were allowed to adhere to 6-well plates in ATCC medium 712 for 30 min at 30°C. The adherent trophozoites were washed and replaced with 500 μl of non-nutrient medium (20 mM Tris-HCl [pH 8.8], 100 mM KCl, 8 mM MgSO_4_, 0.4 mM CaCl_2_ and 1 mM NaHCO_3_). In an Eppendorf tube, 4 μg of Midiprep (PureLink HiPure Midiprep Kit, Thermo Fisher Scientific) plasmid DNA was diluted to 100 μl with non-nutrient medium. Twenty microliters of SuperFect Transfection Reagent (Qiagen Inc, Germantown, MD) was added to the DNA suspension, mixed gently by pipetting five times, and incubated for 10 min at RT. Six hundred microliters of non-nutrient medium were added to the DNA-SuperFect mix, and the entire suspension was added to the trophozoites adhering to the 6-well culture plate. The culture plate was incubated for 3 hr at 30°C, after which the non-nutrient medium was replaced with ATCC medium 712 and incubated for another 24 hr at 30°C. To select for transfectants, we added 12.5 μg/ml of Gibco G418 antibiotic (Thermo Fisher Scientific) to the culture after 24 hr, and we changed the medium plus antibiotic every four days. After 2 to 4 weeks, the transfectants were growing robustly in the presence of the antibiotic, and trophozoites and/or cysts expressing GFP were detected by widefield microscopy. Without prior cloning, transfectants were induced to encyst, fixed after 3 to 144 hr, labeled with WGA and CFW, and examined by widefield microscopy and SIM, as described above.

### Binding of cyst wall proteins fused to MBP or tagged with GFP to microcrystalline cellulose and chitin beads

MBP-fusion constructs were prepared by cloning the cDNA of an abundant Luke(2) lectin (60 to 843-bp CDS of ACA1_377670) and an abundant Jonah(1) lectin (70 to 1599-bp CDS of ACA1_164810) without their signal sequences into pMAL-p2x vector (New England Biolabs) (S1 Excel file) for periplasmic expression in BL21-CodonPlus(DE3)-RIPL (Agilent Technologies, Lexington, MA) [42]. For the MBP-fusion, the Leo CDS without the signal sequence (67 to 564-bp of ACA1_074730) was codon optimized and cloned into pMAL-p2x vector (S1 Excel file). MBP-Luke(2) was induced with 250 μM IPTG for 5 hr at RT; MBP-Jonah(1) was induced with 1 mM IPTG for 5 hr at RT; and MBP-Leo was induced with 250 μM IPTG for 3.5 hr at 37°C. MBP-fusion proteins were purified with amylose resin following the manufacturer’s instructions (GE Healthcare, Pierce, Agilent Technologies, and New England Biolabs). MBP-fusions (1 μg each in 100 μl of 1% NP40) were incubated with 0.5 μg Avicel microcrystalline cellulose (Sigma-Aldrich) or a 50-μl slurry of magnetic chitin beads (New England Biolabs) for 3 hr at 4°C with rocking. Cellulose was centrifuged to collect the supernatant (unbound fraction) and pellet (bound fraction), while chitin beads were collected with a magnet. The bound fractions were washed three times with 1% NP40. To solubilize proteins, the input material (total), unbound (U), and bound (B) fractions were boiled in SDS sample buffer. MBP-proteins were separated on SDS-PAGE gels, blotted to PVDF membranes, blocked in 5% BSA, and detected using anti-MBP antibodies (New England Biolabs).

To test the carbohydrate-binding specificity of the GFP-tagged lectins, we lysed trophozoites expressing Jonah(1) and Luke(2) under a GAPDH promoter and then incubated lysates with microcrystalline cellulose or chitin beads, using methods to characterize MBP-fusions. Total, unbound, and bound proteins were released with SDS, separated on SDS-PAGE, transferred to PVDF, and detected with reagents that recognize GFP. A control was GFP alone expressed under a GAPDH promoter.

### Western blots of *A*. *castellanii* trophozoite and cyst lysates probed with anti-lectin rabbit antibodies

Log-phase trophozoites and 36-hr-old cysts were harvested, and the total protein solubilized in SDS sample buffer, run in SDS-PAGE gels, blotted on PVDF membranes, and blocked in 5% BSA. MBP-cyst wall protein fusions and MBP alone were run in adjacent lanes as positive and negative controls, respectively. The blots were probed with 1:100 dilutions of rabbit polyclonal antibodies (Li International, Denver, Co) raised to 16- or 50-amino acid peptides of abundant Luke(2) lectin (residues 230–279 of ACA1_377670), Leo lectin (residues 124–139 of ACA1_074730) and Jonah(1) lectin (residues 362–411 of ACA1_164810). A 1:1000 dilution of anti-rabbit IgG-HRP (BioRad) was used as secondary antibody and Super Signal West Pico PLUS (Thermo Fisher Scientific) for chemiluminescent detection. Coomassie stained gels were run in parallel for loading control.

### Methods to determine the accessibility of cyst wall proteins and glycopolymers in mature walls and to count ostioles

We used anti-GFP antibodies to determine the accessibility of GFP-tagged lectins in mature cyst walls. Without prior fixation, mature cysts expressing GFP-fusions under their own promoter were blocked with 1% BSA, incubated with 1:400 mouse anti-GFP IgG (Roche) for one hr at RT, washed, and then incubated with 1:800 goat anti-mouse IgG-Alexa Fluor 594 (Molecular Probes, Invitrogen). Preparations were washed, labeled with WGA and CFW, fixed in paraformaldehyde, mounted on glass slides, and observed with widefield microscopy, as described above. To determine the accessibility of glycopolymers in mature cyst walls, we used MBP-fusions to Luke(2), Leo, and Jonah(1) lectins. Mature cysts were fixed, blocked, and incubated with 15 μg of each MBP-cyst wall protein fusion conjugated to Alex Fluor 594 for 2 hr at 4°C. Preparations were labeled with WGA conjugated to Alexa Fluor 488 and CFW, as described above, and visualized with widefield microscopy and SIM. To count the number of ostioles per cyst wall, we rotated three-dimensional SIM reconstructions of mature cysts expressing Luke(2)-GFP or Leo-GFP or non-transfectants labeled with WGA, MBP-Luke(2), or MBP-Leo, all of which clearly outlined conical ostioles.

## Results

### TEM and SIM showed purified *A*. *castellanii* cyst walls contained distinct endocyst and ectocyst layers, as well as ostioles

**Fig 1:**
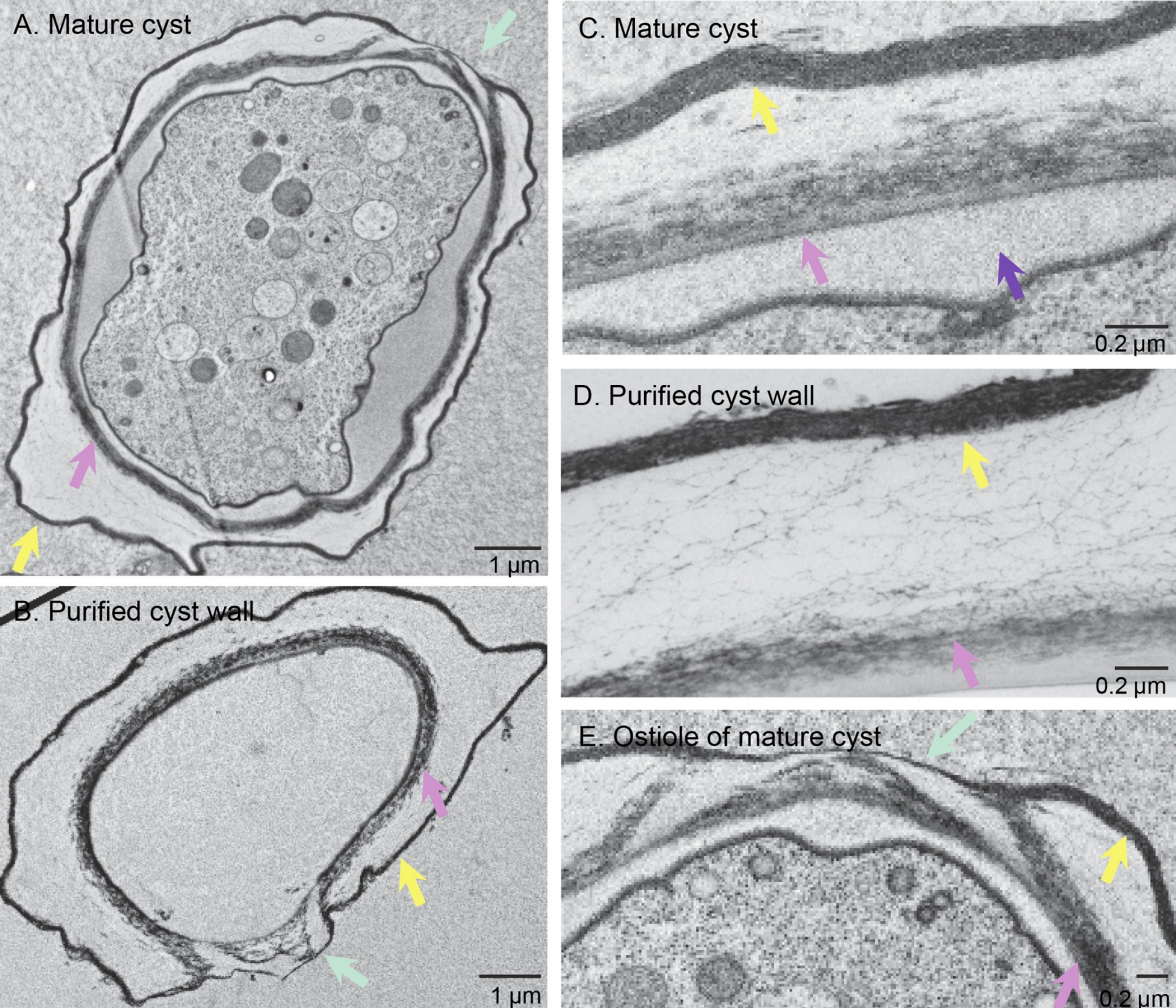
TEM showed purified *A*. *castellanii* cyst walls retained endocyst and ectocyst layers and ostioles. A, B. A mature cyst and a purified cyst wall had an outer ectocyst layer (yellow arrows), an inner endocyst layer (pink arrows), and ostioles (turquoise arrows) that connect the layers. Endocyst and ectocyst layers had the same appearance in mature cysts (C) and purified cyst walls (D). Purified cyst walls were missing amorphous material (purple arrow) between the wall and the plasma membrane of mature cysts. E. At the edge of the ostiole of a mature cyst, the endocyst layer bifurcated, and the outer branch met the ectocyst layer. In the center of the ostiole, the ectocyst layer formed a narrow cap over the inner branch of the endocyst layer. Scale bars as marked on micrographs.

Cyst wall preparations were made by subjecting mature cysts to sonication (for TEM) or bead beating (for SIM), followed by density centrifugation to remove cellular contents. For TEM, mature cysts and purified cyst walls were frozen under high pressure, and fixatives were infiltrated at low temperature [48]. Purified cyst walls had intact ectocyst and endocyst layers, as well as conical ostioles that link them (Fig 1) [20]. The purified walls were missing amorphous material that fills the space between the inner aspect of the cyst wall and the plasma membrane of the trophozoite inside.

**Fig 2:**
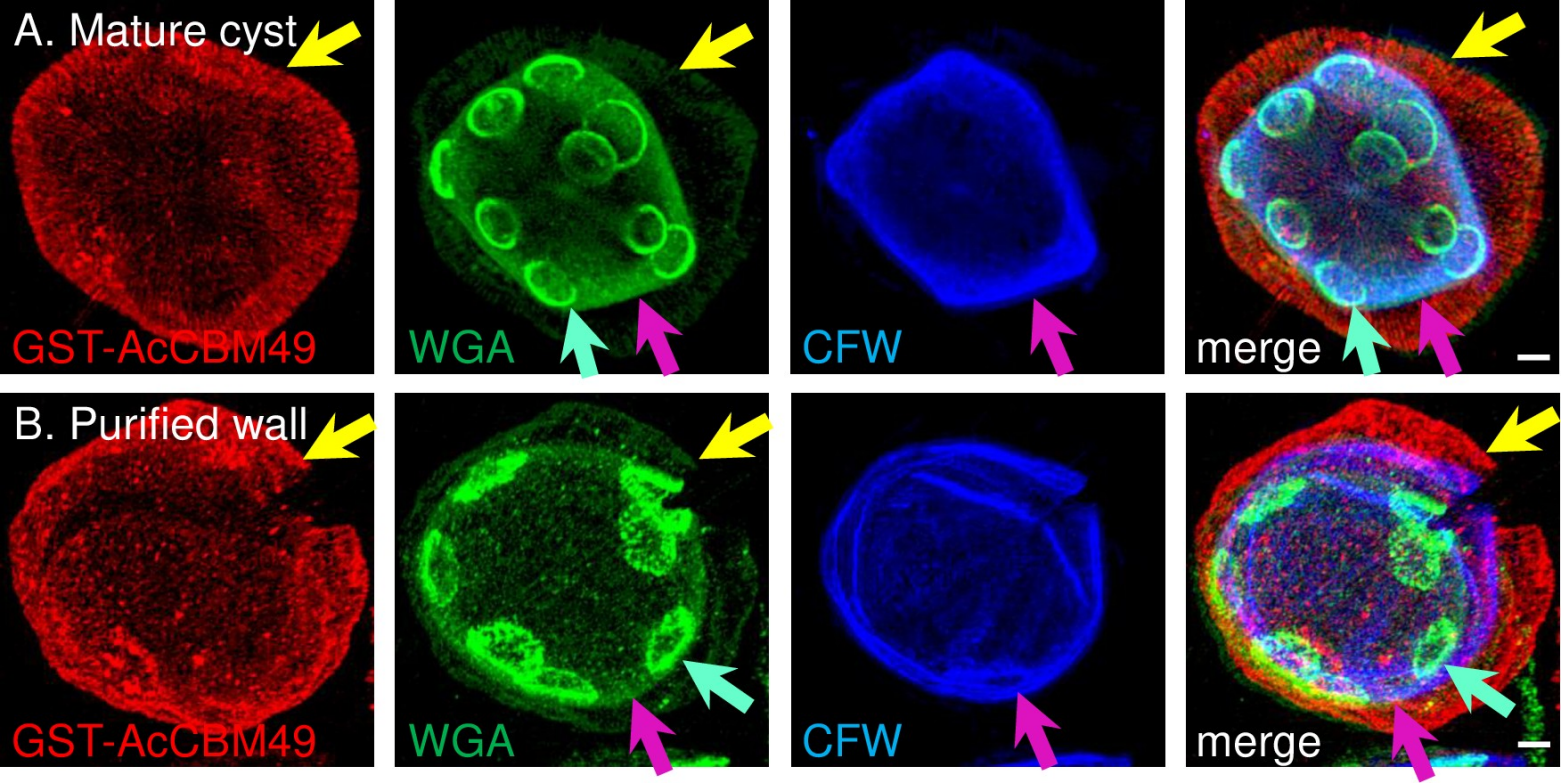
SIM showed purified *A*. *castellanii* cyst walls retained distinct ectocyst layer and endocyst layer, as well as ostioles. The ectocyst layer (yellow arrows) of a mature cyst (A) and purified cyst wall (B) labeled red with GST-AcCBM49; the edges of ostioles (turquoise arrows) labeled green with WGA; and the endocyst layer (pink arrows) labeled blue with CFW. WGA also labeled less strongly the endocyst and ectocyst layers. While it appears that some ostioles overlap each other, rotation of the deconvolved images showed they are actually on opposite sides of the spherical surface. Scale bars are 2 μm.

For SIM, we used probes that bind chitin (WGA) and β-1,3 and β-1,4 polysaccharides (CFW) in the walls of fungi and cysts of *Entamoeba* [44–46]. CFW, a fluorescent brightener, has also been used to diagnose *Acanthamoeba* cysts in eye infections [47]. In addition, we made a glutathione-S-transferase (GST) fusion-protein, which contains the N-terminal CBM49 of a candidate cyst wall protein of *A*. *castellanii* (S1 Fig and S1 Excel file) [43]. The GST-AcCBM49 expression construct was designed to replicate that used to determine the carbohydrate binding properties of SlCBM49, the C-terminal carbohydrate-binding module of the *S*. *lycopersicum* cellulase SlGH9C [33]. In both mature cysts and purified cyst walls, GST-AcCBM49 predominantly labeled the ectocyst layer, WGA highlighted the ostioles, and CFW labeled the endocyst layer (Fig 2). A detailed examination of both the mature cyst and the purified wall showed WGA also labeled the endocyst layer and the ectocyst layer (weakly).

In summary, TEM and SIM both showed that ectocyst and endocyst layers, as well as ostioles, were intact in purified cyst walls, which were relatively free of cellular material. While GST-CBM49, WGA, and CFW, as well as abundant cyst wall proteins (see below), were extremely useful for distinguishing structures in the developing and mature cyst walls, their lack of carbohydrate-binding specificity (again see below) made it impossible to distinguish whether ectocyst layer, endocyst layer, and ostioles were composed of cellulose, chitin, or both glycopolymers. Indeed, nowhere in this paper have we shown that chitin or chitosan are present in cyst walls of *A*. *castellanii*.

### Mass spectrometry showed candidate cyst wall proteins of *A*. *castellanii* were encoded by multigene families and contained tandem repeats of short domains

Trypsin treatment of purified *A*. *castellanii* cyst walls, which was followed by LC-MS/MS of the released peptides, gave similar results in five biological experiments (Table 1 and S2 Excel file). While some proteins remained in cyst walls after trypsin digestion, their identities were similar to those detected in the soluble fractions by in gel-digests with trypsin or chymotrypsin. Candidate cyst wall proteins with the most unique peptides identified by LC-MS/MS belonged to three families, which we named Luke, Leo, and Jonah lectins, because each bound to cellulose +/- chitin (see below). Although it was impossible to draw a line that separates actual cyst wall proteins from contaminating proteins, secreted proteins with 18+ unique peptides included six Leo lectins, four Luke lectins, and three Jonah lectins. The vast majority of proteins with <18 unique peptides were predicted to be cytosolic (including CSP21) and so were likely intracellular contaminants of cyst wall preparations. The exception to this hypothesis, we think, were additional Luke, Leo, and Jonah lectins, which were most likely less abundant cyst wall proteins. For readers interested in cytosolic proteins of *A*. *castellanii*, we have added S3 Excel file, which contains all the mass spectrometry data, which included a “dirty” cyst wall preparation that was generated without using the Percoll gradient or porous filter.

**Table 1:** Candidate cyst wall proteins of *A*. *castellanii* identified by mass spectrometry.

	ID	# Unique peptides	Coverage (%)	Mass (kDa)
Jonah lectins				
three CAAs	ACA1_157320	147	38	146
one CAA	ACA1_164810	83	56	58
	ACA1_261530	18	23	55
	ACA1_133400	9	24	44
	ACA1_377440	6	11	47
Luke lectins				
three CBM49s	ACA1_245650	72	74	44
	ACA1_160160	8	25	43
	ACA1_187760	7	25	42
	ACA1_252830	6	19	44
	ACA1_031530	6	21	43
	ACA1_253650	5	20	42
	ACA1_253500	5	19	42
	ACA1_061050	3	12	43
	ACA1_287530	2	14	43
two CBM49s	ACA1_377670	78	68	29
	ACA1_096300	47	77	28
	ACA1_246110	22	70	27
Leo lectins				
two 8-Cys domains	ACA1_074730	34	82	20
	ACA1_351320	24	44	20
	ACA1_394030	24	44	20
	ACA1_394280	24	36	24
	ACA1_083920	19	68	20
	ACA1_394560	1	10	19
two 8-Cys + TKH	ACA1_188350	21	20	59
	ACA1_374130	7	20	52
	ACA1_188550	7	15	46
	ACA1_188370	6	9	68
	ACA1_116240	5	18	56
	ACA1_365840	3	18	44
	ACA1_117050	3	33	36
	ACA1_096640	2	27	37

**Fig 3:**
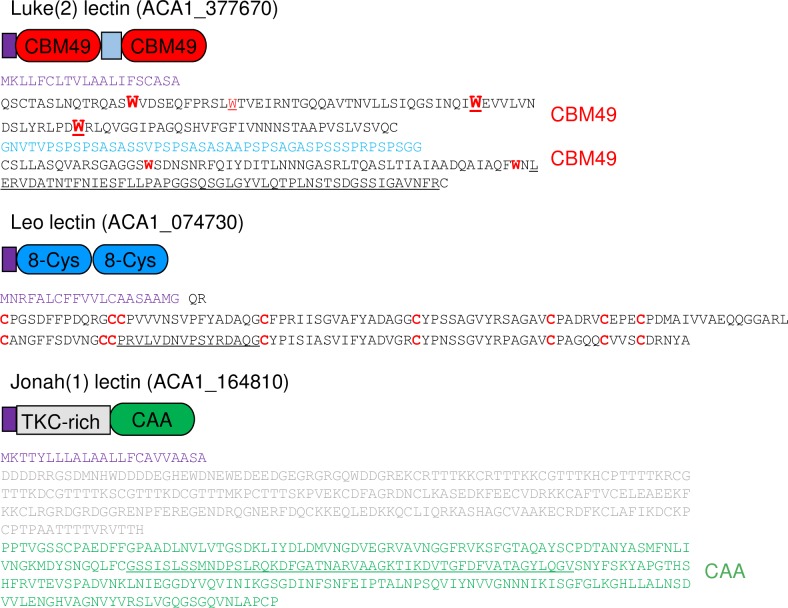
Abundant cyst wall proteins contained two CBM49s (Luke(2) lectin), two 8-Cys domains (Leo lectin), or one CAA domain (Jonah(1) lectin). The Luke(2) lectin had an N-terminal signal peptide (purple) and two CBM49s separated by short Ser- and Pro-rich spacers (light blue). The N-terminal CBM49 contained four Trp residues (red Ws), three of which were conserved in a C-terminal CBM49 of a tomato cellulase (larger font) and three of which were conserved in a single CBM49 of *Dictyostelium* cellulose-binding protein (XP_629733) (underlined). In contrast, the C-terminal CBM49 had two conserved Trp residues. The Leo lectin had a signal peptide and two unique domains (dark blue) containing eight Cys residues each (red Cs). The Jonah(1) lectin had a signal peptide, a Thr-, Lys-, and Cys-rich domain (gray), and a single CAA domain (green). Peptides used to immunize rabbits are underlined. An abundant Luke(3) lectin with three CBM49s, Leo(TKH) lectin with a Thr-, Lys-, and His-rich spacer, and an abundant Jonah(3) lectin with three CAA domains are shown in S2 Fig.

Luke lectins were comprised of an N-terminal signal peptide, followed by two or three CBM49s that were separated by Ser- and Pro-rich spacers (Fig 3 and S2 Fig) [33, 34, 50]. The N-terminal CBM49 of Luke lectins contained three conserved Trp resides conserved in SlCBM49 from tomato. A fourth conserved Trp is present in the CBM49 of *D*. *discoideum* cellulose-binding proteins [56]. The other CBM49s (middle and/or C-terminal) of Luke lectins had two conserved Trp residues. Luke lectins were acidic (pI 5 to 6) and had formula weights (FWs) from 27 to 29-kDa (two CBM49s) or 42 to 44-kDa (three CBM49s). There were no predicted transmembrane helices or glycosylphosphatidylinositol anchors in the Luke or Leo lectins [51, 52]. LC-MS/MS of the released cell wall peptides identified at least one unique peptide corresponding to all 12 genes encoding Luke lectins, although the number of unique peptides varied from 78 to two (Table 1 and S2 Excel file). In general, Luke lectins with two CBM49s had more unique peptides than Luke lectins with three CBM49s. One to four unique peptides were derived from three CBM49-metalloprotease fusion-proteins, which consisted of an N-terminal signal peptide followed by a single CBM49 with four conserved Trp residues and a metalloprotease (ADAM/reprolysin subtype) with a conserved catalytic domain (HEIGHNLGGNH) [53]. We used an abundant Luke(2) lectin (ACA1_377670) with two CBM49s to perform RT-PCR, make rabbit anti-peptide antibodies, and make maltose-binding protein (MBP)- and green fluorescent protein (GFP)-fusions (Fig 3 and S1 Fig) [40–42]. We also used an abundant Luke(3) lectin (ACA1_245650) with three CBM49s to make a GFP-fusion (S2 Fig).

Leo lectins were comprised of an N-terminal signal peptide, followed by two repeats of a unique 8-Cys domain, some of which were separated by a long Thr-, Lys-, and His-rich spacer (Fig 3 and S2 Fig). Leo lectins without a spacer were acidic (pI ~4.8) and had FWs from 19 to 24-kDa, while Leo lectins with the TKH-rich spacer were basic (pI ~8.3) and had FWs from 36- to 59-kDa. Leo lectins were encoded by 16 genes, of which 14 proteins were identified by our LC-MS/MS analysis. While the number of unique peptides varied from 34 to one, Leo lectins without a spacer generally had more unique peptides than Leo lectins with the TKH-rich spacer. We used abundant Leo lectin without a spacer (ACA1_074730) to perform RT-PCR, make rabbit anti-peptide antibodies, and make MBP- and GFP-fusions (S1 Fig).

Jonah lectins were comprised of an N-terminal signal peptide followed by one or three choice-of-anchor A (CAA) domains (Fig 3 and S2 Fig) [53]. The binding activity of the CAA domain, which is adjacent to a collagen-binding domain in a microbial surface component recognizing the adhesive matrix molecule (MSRAMM) of *Bacillus anthracis*, was not characterized [57]. Jonah(1) lectins with a single CAA domain were acidic (pI ~6), had a FW from 44 to 58-kDa and had an N-terminal Thr-, Lys-, and Cys-rich domain. A Jonah(3) lectin with three CAA domains was basic (pI ~8.8), had a FW of ~146-kDa, and contained Ser- and Pro-rich spacers between CAA domains, as well as hydrophobic regions that may be transmembrane helices [51]. Jonah lectins were encoded by eight genes, of which five were identified by our LC-MS/MS analysis based on one to 147 unique peptides. We used an abundant Jonah(1) lectin (ACA1_164810) with a single CAA domain to perform RT-PCR, make rabbit anti-peptide antibodies, and make MBP- and GFP-fusions (S1 Fig).

Other secreted proteins with 18+ unique peptides detected by LC-MS/MS, which are candidate cyst wall proteins, included a laccase with three copper oxidase domains (ACA1_068450), a protein with a C-terminal ferritin-like domain (ACA1_292810), a Kazal-type serine protease inhibitor (ACA1_291590), a conserved uncharacterized protein (ACA1_068630), and a protein unique to *A*. *castellanii* (ACA1_145900) [53, 54, 58]. Interestingly, a bacterial laccase has been shown to bind cellulose [59]. There were also three serine proteases, which have been localized to the secretory system of encysting *A*. *castellanii* [60].

These results suggested that the most abundant candidate cyst wall proteins of *A*. *castellanii* contain tandem repeats of conserved domains (CBM49 in Luke lectins and CAA in Jonah lectins) or a unique domain (8-Cys in Leo lectins). Peptides corresponding to nearly all members of each gene family were detected by mass spectrometry. However, the relative abundances of unique peptides for each cyst wall protein varied by more than an order of magnitude, suggesting marked differences in gene expression. Because it was not possible to separate cyst walls into component parts (endocyst and ectocyst layers and ostioles) prior to LC-MS/MS analysis of tryptic peptides, we used SIM and GFP-tags to localize abundant members of each protein family in cyst walls of transfected *A*. *castellanii* (see below).

### Origins and diversity of genes that encode Luke, Leo, and Jonah lectins

Leo lectins, which had two domains with 8-Cys each, appeared to be unique to *A*. *castellanii*, as no homologs were identified when BLAST analysis were performed using the nonredundant (NR) database at NCBI (https://www.ncbi.nlm.nih.gov/) [35]. The origin of genes encoding Luke lectins was difficult to infer, because its CBM49s showed only a 31% identity over a short (77-amino acid) overlap with a predicted cellulose-binding protein of *D*. *discoideum* (expect value of BLASTP was just 7e-05) [33, 34, 56]. In contrast, the CAA domain of Jonah lectins appeared to derive from bacteria by horizontal gene transfer (HGT), as no other eukaryote contained CAA domains, and there was a 28% identity over a bigger (263-aa) overlap with a choice-of anchor A family protein of *Saccharibacillus sp*. *O16* (5e-12) [35, 53]. The *A*. *castellanii* laccase (also known as copper oxidase), whose signals were abundant in the mass spectra, was likely the product of HGT from bacteria, as there was a 44% identity over a large (526-aa) overlap with a copper oxidase of *Caldicobacteri oshimai* (6e-135) [58]. The uncertainty was based upon the presence of similar enzymes in plants, one of which (*Ziziphus jujube*) showed a 39% identity over a 484-aa overlap (4e-101) with the *A*. *castellanii* laccase.

No pairs of genes within each lectin family were syntenic as defined by AmoebaDB, indicating duplicated genes are paralogs [36]. With the exception of two Luke lectins (ACA1_253500 and ACA1_253650) that were 98% identical and two Leo lectins (ACA1_074770 and ACA1_083920) that were 85% identical, members of each family of cyst wall proteins differed in amino acid sequence by >40%. Genes that encode cyst wall proteins also varied in the number of introns (zero to two in Luke, two to four in Leo, and zero to 24 in Jonah). Searches of genomic sequences of 11 strains of *Acanthamoebae*, deposited in AmoebaDB without protein predictions by Andrew Jackson of the University of Liverpool, using TBLASTN and sequences of abundant Luke, Leo, and Jonah lectins localized in the next section, showed four results [35, 36]. First, although stop codons were difficult to identify using this method, all 11 strains appeared to encode each cyst wall protein. Second, most strains showed 100 to 200-amino acid stretches of each cyst wall protein that were 80 to 90% identical to the *A*. *castellanii* Neff strain studied here. These stretches did not include low complexity spacers, which were difficult to align. Third, some of the strains showed greater differences from the Neff strain in each cyst wall protein, consistent with previous descriptions of *Acanthamoeba* strain diversity based upon 18S rDNA sequences [61]. Fourth, while coding sequences and 5’ UTRs were well-conserved, intron sequences were very poorly conserved, with the exception of branch-point sequences.

In summary, genes encoding Jonah lectins and laccase likely derived by HGT, while genes encoding Leo lectins appeared to originate within *Acanthamoeba*. Although CBM49s of Luke lectins shared common ancestry with plants and other Amoebazoa, their precise origin was not clear. For the most part, gene duplications that expanded each family within the *Acanthamoeba* genome occurred a long time ago, as shown by big differences in amino acid sequences of paralogous proteins and variations in the number and sequences of introns. Regardless, the set of Luke, Leo, and Jonah lectins identified by mass spectrometry, as well as the sequences of abundant cyst wall proteins localized in the next section, appeared to be conserved among 11 sequenced isolates of *Acanthamoebae*.

### In the first stage of encystation (3 to 9 hr), a Jonah(1) lectin and glycopolymers labeled by WGA and GST-CBM49 were present in distinct vesicles

To localize candidate cyst wall proteins, we expressed an abundant Leo lectin with no spacer and an abundant Jonah(1) lectin with a single CAA domain, each with a GFP-tag under its own promoter (446- and 571-bp of the 5’ UTR, respectively) in transfected trophozoites of *A*. *castellanii*, using an episomal vector that was selected with G418 (S1 Fig) [40, 41]. We also expressed an abundant Luke(2) lectin with two CBM49s and an abundant Luke(3) lectin with three CBM49s, each with a GFP-tag under its own promoter (486- and 500-bp of the 5’ UTR, respectively). GFP-tagged candidate cyst wall proteins expressed under their own promoter were absent in the vast majority of log-phase trophozoites, while GFP-tagged cyst wall proteins were present in small numbers in trophozoites in stationary cultures, where a few organisms began to encyst spontaneously.

**Fig 4:**
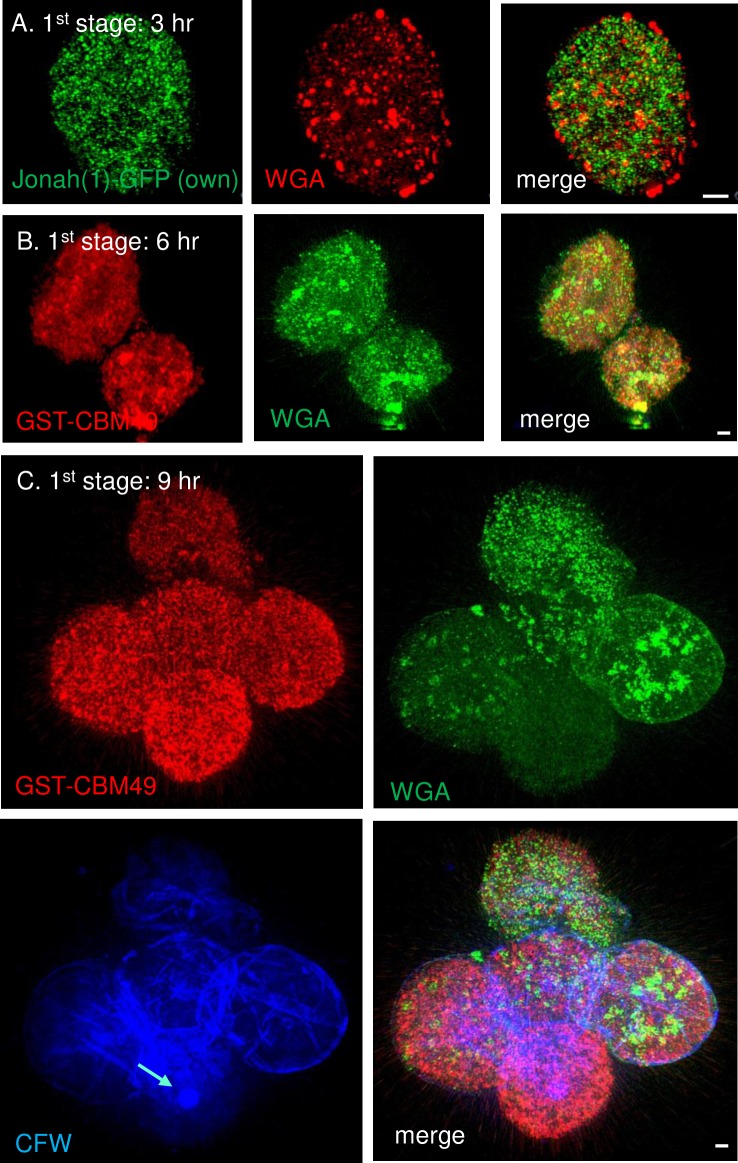
During the first stage of encystation, SIM showed Jonah(1)-GFP and glycopolymers labeled by WGA and GST-CBM49 were present in dozens of distinct vesicles. A. After 3 hr encystation, Jonah(1)-GFP (green), which was expressed under its own promoter, was present in dozens of small vesicles. WGA (red) labeled fewer but larger vesicles, which did not overlap with those containing Jonah(1)-GFP (see merge). B. After 6 hr encystation, glycopolymers labeled by GST-CBM49 (red) were present in dozens of vesicles that did not overlap with those labeled by WGA (green). C. After 9 hr encystation, glycopolymers labeled with GST-CBM49 and WGA were again very abundant in vesicles that did not overlap. CFW was not visible in vesicles of organisms encysting for 3 to 6 hr, but CFW (blue) labeled the surface of encysting protists at 9 hr. A single ostiole, which was small and circular (turquoise arrow), was present on the surface of one encysting protist, while ostioles were absent from the other organisms. When expressed under its own promoter, neither Luke(2)-GFP nor Leo-GFP was present in the first stage of encystation. A to C. Scale bars are 2 μm.

As early as three hours after placement on non-nutrient agar, Jonah(1)-GFP expressed under its own promoter was present in dozens of small vesicles (Fig 4A). The glycopolymer detected with WGA was also made early and was present in vesicles of varying sizes, which did not overlap with those containing Jonah(1)-GFP. The glycopolymer labeled with GST-CBM49 was also made early in dozens of small vesicles, which were distinct from those labeled with WGA (Fig 4B and 4C). Glycopolymers labeled with CFW were not visible in organisms encysting for 3 and 6 hr, but CFW labeled a thin, spherical wall after 9 hr encystation. At this time, rare protists had one or two small, flat ostioles, but most organisms had none. Finally, neither Luke(2)-GFP nor Leo-GFP, each expressed under its own promoter, was visible during this first stage of development of the cyst wall.

These results showed that the first stage of encystation is an abrupt event in which amoeboid trophozoites rapidly synthesize glycopolymers and a Jonah(1) lectin in dozens of vesicles that fill the encysting cells. In contrast, Luke(2) and Leo lectins were not yet made, suggesting encystation-specific proteins are expressed at different times [28, 62].

### In the second stage of encystation (12 to 18 hr), a thin primordial cyst wall contained small flat ostioles and three abundant lectins in distinct distributions

**Fig 5:**
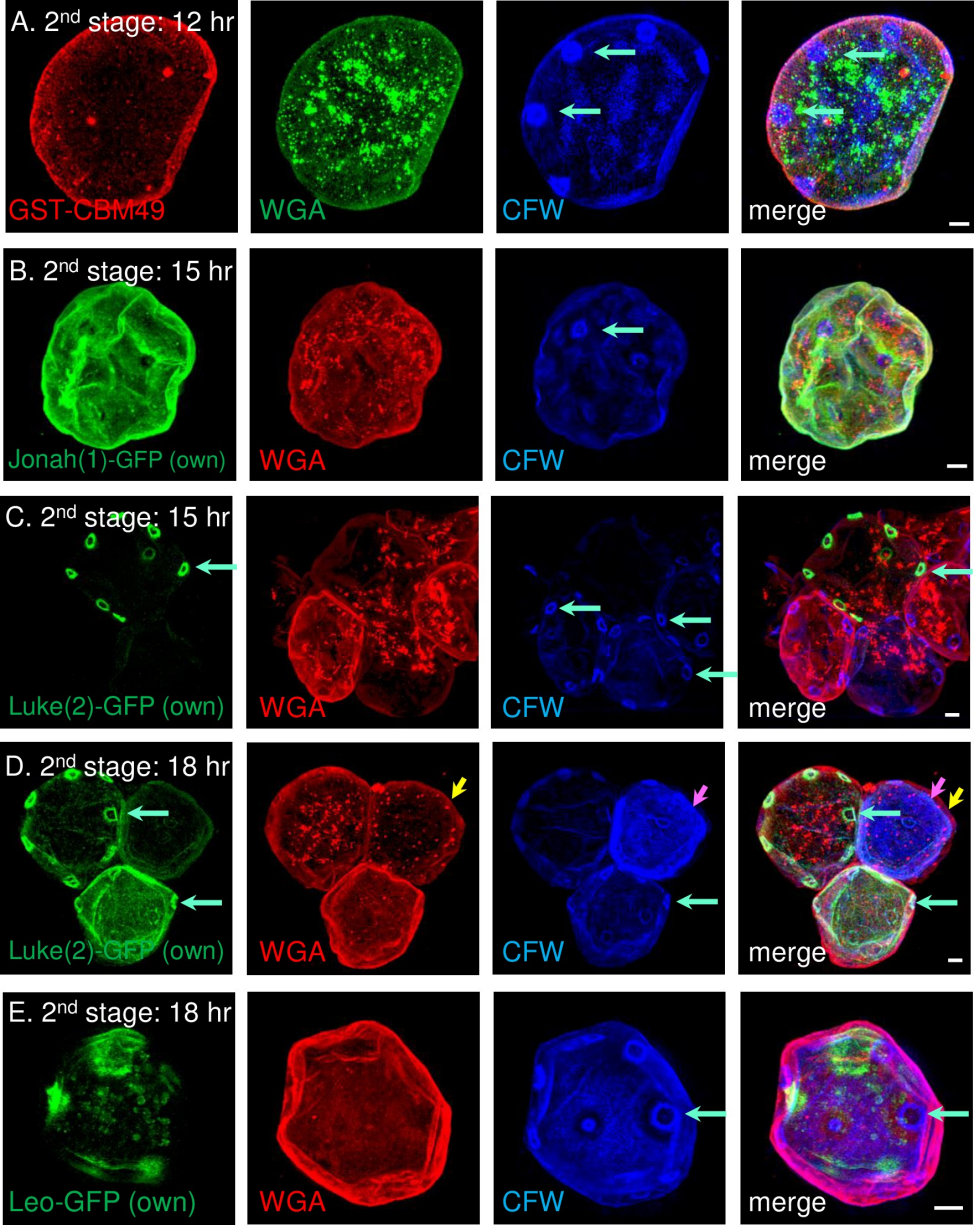
During the second stage of encystation, SIM showed a primordial cyst wall was comprised of Jonah(1)-GFP and glycopolymers labeled with GST-CBM49 and WGA, each in a diffuse pattern, while small, flat ostioles were outlined by Luke(2)-GFP and labeled with CFW. A. After 12 hr encystation, GST-CBM49 (red) diffusely labeled a thin, primordial wall, which contained small, flat ostioles (turquoise arrows) visible only with CFW (blue). WGA (green), which predominantly labeled vesicles, also labeled the thin, primordial wall. After 15 hr encystation, Jonah(1)-GFP (green), expressed under its own promoter, was homogenously distributed in the primordial wall (B), while Luke(2)-GFP (green), also expressed under its own promoter, outlined the edges of small ostioles in some cells (C). After 18 hr encystation, Luke(2)-GFP, which continued to outline the edges of small ostioles, also spread across the surface of some primordial walls (D), while Leo-GFP, expressed under its own promoter, was in a patchy distribution in primordial walls that was, for the most part, independent of ostioles (E). Also at 18 hr in some cells, there were the beginnings of an outer ectocyst layer (yellow arrow) and an inner endocyst layer (pink arrows). A to E. Scale bars are 2 μm.

GST-CBM49, WGA, and CFW each labeled primordial cyst walls, which had a single, thin layer and small, flat ostioles (Fig 5A). Ostioles, which labeled with CFW but not with GST-CBM49 or WGA, were at first filled circles but later became rings (Fig 5B). While it was difficult to count these small ostioles because of variable labeling with CFW, they appeared to be in similar number and distribution as conical ostioles of mature cyst walls (see below). Each of the GFP-tagged lectins expressed under its own promoter was present in primordial cyst walls but in markedly different distributions. Jonah(1)-GFP was homogenously distributed across the surface of the primordial cyst wall (Fig 5B). Luke(2)-GFP outlined some but not all of early ring-shaped ostioles (Fig 5C). Later, in addition to outlining the ostioles, Luke(2)-GFP was homogenously distributed across the surface of the primordial cyst wall (Fig 5D). Leo-GFP was latest to the wall and had a patchy distribution, which was, for the most part, independent of the ostioles (Fig 5E).

These results showed that in the second stage of encystation the primordial cyst walls contained three abundant lectins, each in a distinct distribution. The presence of small, circular ostioles, which were visualized by the external probe CFW or the internal probe Luke(2)-GFP, showed these structures are initiated prior to separation of the ectocyst and endocyst layers.

### In the third stage of encystation (24 to 36 hr), the ectocyst and endocyst layers separated, the ostioles became dome-shaped, and the three lectins moved towards their positions in mature cyst walls

**Fig 6:**
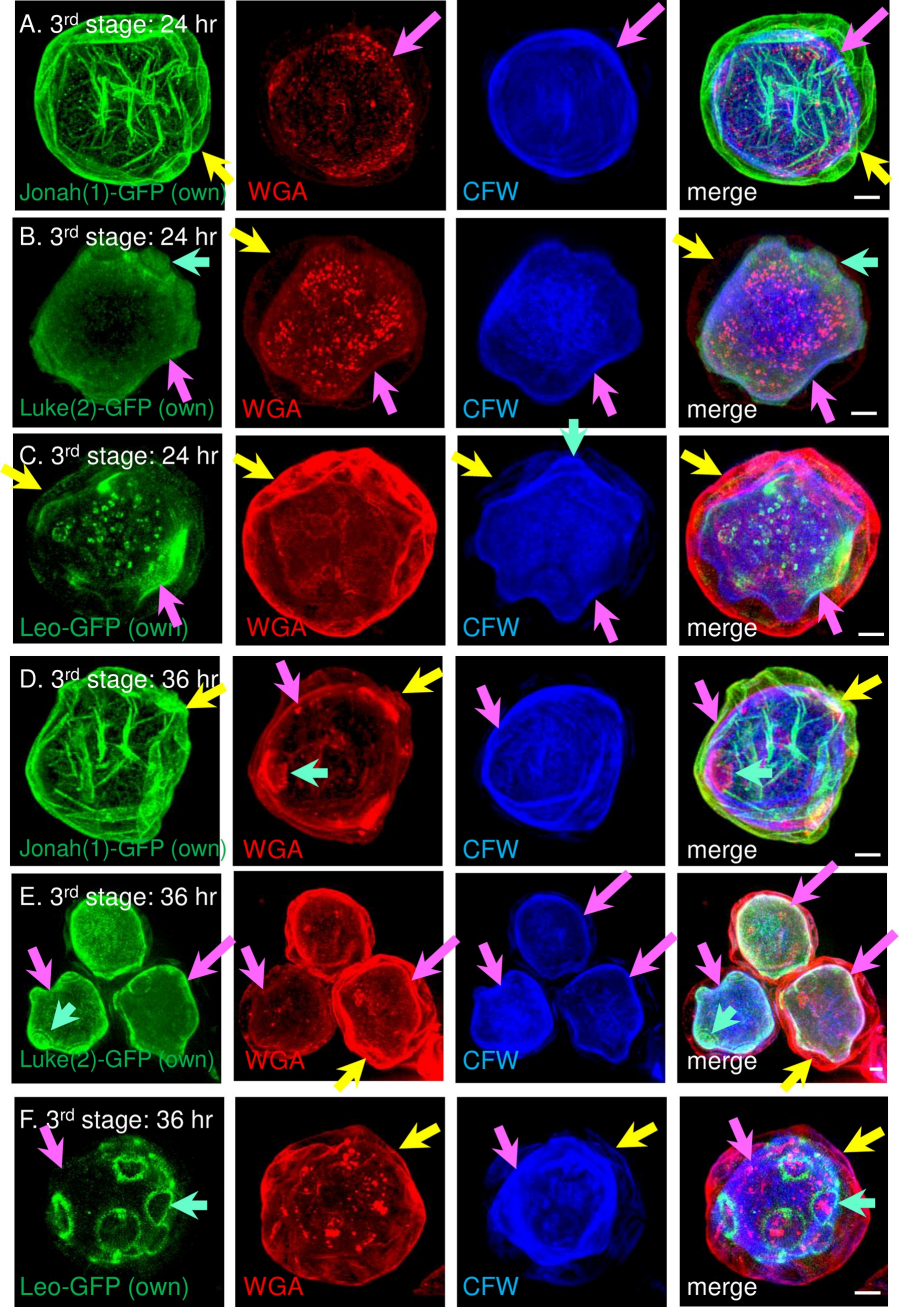
During the third stage of encystation, SIM showed Jonah(1)-GFP remained in the ectocyst layer, while Luke(2)-GFP and Leo-GFP moved to the endocyst layer and ostioles. Each of the GFP-tagged lectins was expressed under its own promoter. Jonah(1)-GFP (green) was abundant in the ectocyst layer (yellow arrows) of walls of protists encysting for 24 hr (A) or 36 hr (D). Luke(2)-GFP was homogeneously distributed in the endocyst layer (pink arrows), as well as dome-shaped ostioles (turquoise arrows), at 24 hr (B) and at 36 hr (E) encystation. Leo-GFP was present in vesicles and in a somewhat patchy distribution in both the ectocyst and endocyst layers of organisms encysting for 24 hr (C). It was not until 36 hr (F) that Leo-GFP began to sharply outline ostioles. CFW (blue) consistently labeled the endocyst layer and occasionally labeled the ostioles (C) or ectocyst layer (F). WGA (red) labeled the endocyst layer (A), the ectocyst layer (C and F), or both layers (B, D, and E). A to F. Scale bars are 2 μm.

In the third stage, the cell body contracted, so that the emerging endocyst layer was made inside the ectocyst layer (Fig 6). Glycopolymers labeled by CFW moved to the endocyst layer, which was labeled in a variable manner by WGA. Jonah(1)-GFP remained with the ectocyst layer and had essentially the same appearance in the walls of second and third stage cysts (Figs 5B, 6A and 6D). Luke(2)-GFP was diffusely distributed in the endocyst layer and dome-shaped ostioles of organisms encysting for 24 and 36 hr (Fig 6B and 6E). For the most part, Leo-GFP localized the endocyst layer of 24 hr cysts, although its distribution remained patchy (Fig 6C). It was not until 36 hr encystation that Leo-GFP began to diffusely label the endocyst layer and outline ostioles (Fig 6F).

In summary, during the third stage of encystation, Jonah(1)-GFP remained in the outer layer of the wall, which is destined to become the ectocyst layer of mature cyst walls (see next section). In contrast, Luke(2)-GFP and Leo-GFP moved to the inner layer of the wall, which will become the endocyst layer and ostioles of mature cyst walls.

### Jonah(1) lectin was abundant in the ectocyst layer, while Luke(2) and Leo lectins were abundant in endocyst layer and ostioles of mature cyst walls independent of the timing of lectin gene expression

**Fig 7:**
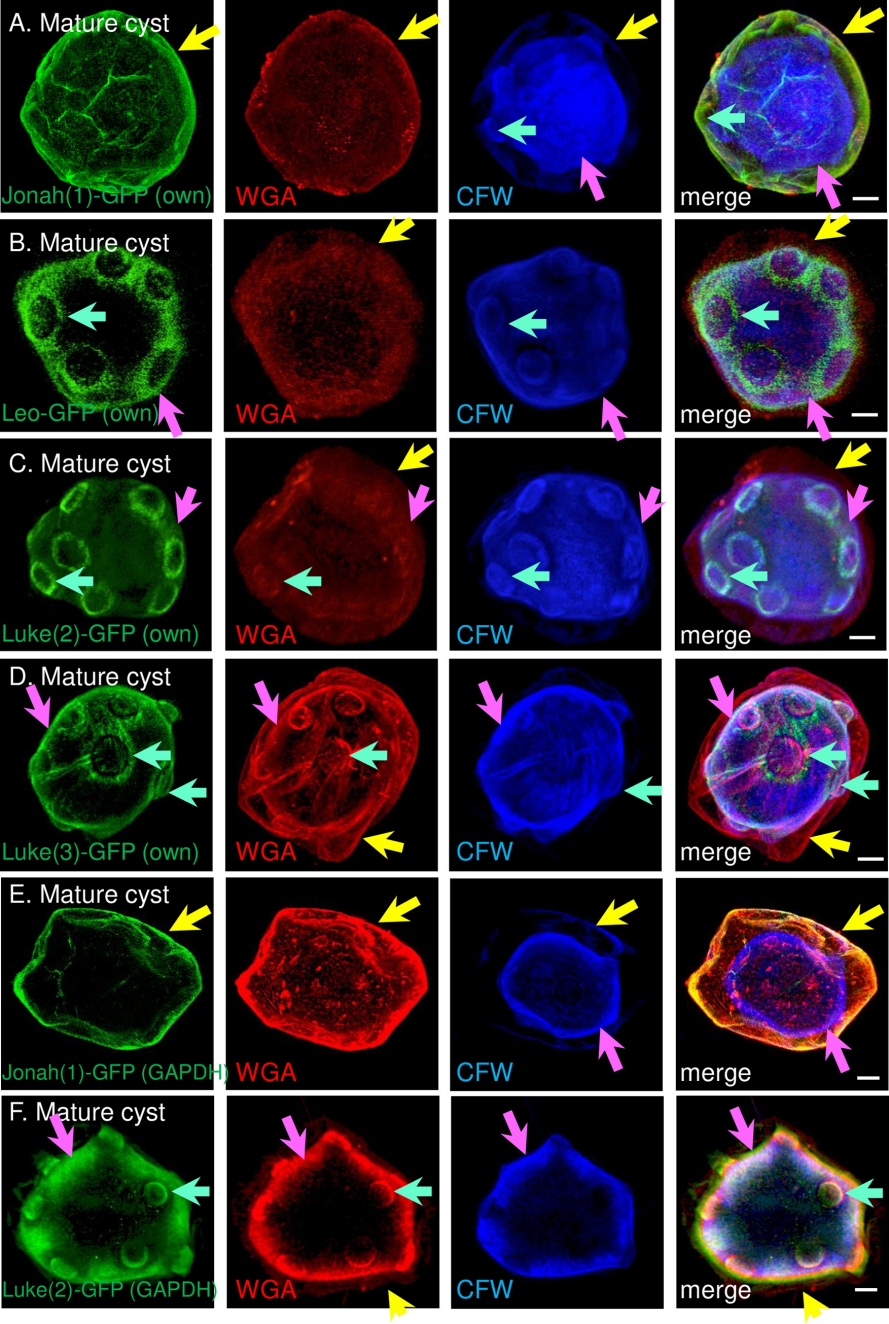
SIM showed an abundant Jonah(1) lectin localized to the ectocyst layer of mature cyst walls, while abundant Luke(2), Luke(3), and Leo lectins localized to the endocyst layer and ostioles. A. A Jonah(1) lectin with a single CAA domain, which was tagged with GFP and expressed under its own promoter in transfected *A*. *castellanii*, localized to the ectocyst layer (yellow arrows) of the wall of mature cysts. WGA labeled both ectocyst and endocyst layers, while CFW labeled the endocyst layer (pink arrows) and ostioles (turquoise arrows). B. Leo-GFP with two 8-Cys domains, which was also expressed under its own promoter, was present in the endocyst layer and sharply outlined the ostioles. Luke(2)-GFP with two CBM49s (C) and Luke(3) with three CBM49s (D), each expressed under its own promoter, were also present in the endocyst layer and outlined conical ostioles of mature cysts. When expressed under a GAPDH promoter, Jonah(1)-GFP localized to the ectocyst layer (E), while Luke(2)-GFP localized to the endocyst layer and ostioles (F). CFW (blue) consistently labeled the endocyst layer, often labeled the ostioles (A to D), and rarely labeled the ectocyst layer (A and E). In this experiment, WGA (red) consistently labeled the ectocyst layer, often labeled the endocyst layer (C, D, and F), and often labeled ostioles (C, D, and F). A to F. Scale bars are 2 μm.

Jonah(1)-GFP expressed under its own promoter was present in the ectocyst layer of mature cyst walls (≥ 36 hr encystation), which were labeled with WGA and CFW (Fig 7A). In contrast, Leo-GFP, Luke(2)-GFP, and Luke(3)-GFP, each expressed under its own promoter, were present in the endocyst layer and sharply outlined the ostioles (Fig 7B to 7D). Jonah(1)-GFP expressed under a constitutive GAPDH promoter localized to the ectocyst layer of mature walls (Fig 7E), while Luke(2)-GFP expressed under the GAPDH promoter localized to endocyst layer and ostioles of mature walls (Fig 7F). Because Leo-GFP did not express well under the GAPDH promoter, it was not possible to compare its distribution versus Leo-GFP under its own promoter.

These results suggested carbohydrate-binding specificities or protein-protein interactions were more important than timing of expression for localization of Jonah(1) and Luke(2) lectins. While the Jonah(1) lectin localized to the ectocyst layer, Luke and Leo lectins, which do not share common ancestry, both localized to the endocyst layer and ostioles. Finally, Luke lectins with either two or three CBM49s localized to the same place.

Numerous control experiments suggested the timing of expression and locations of the GFP-tagged cyst wall proteins in cyst walls were accurate. First, RT-PCR showed that mRNAs of abundant Luke(2), Leo, and Jonah(1) lectins, as well as cellulose synthase (ACA1_349650), were absent or nearly absent from trophozoites but were present during the first three days of encystation (S3 Fig). In contrast, glyceraldehyde 3-phosphate dehydrogenase (GAPDH), which catalyzes the sixth step in glycolysis, was expressed by both trophozoites and encysting *A*. *castellanii* [41]. Second, monospecific, polyclonal rabbit antibodies to a 50-amino acid peptide of an abundant Jonah(1) lectin and a 16-amino acid peptide of an abundant Leo lectin bound to Western blots of proteins from cysts but not from trophozoites (S4 Fig). We were unable to generate rabbit antibodies to the Luke(2) lectin, using methods that worked to make antibodies to Jonah(1) and Leo lectins. Because the rabbit anti-peptide antibodies failed to recognize native proteins, none was useful for localizing cyst wall proteins by SIM. Third, GFP-tagged CSP21 expressed under its own promoter was present in cytosolic accumulations of mature cysts (S5 Fig) [28, 62]. As CSP21 is homologous to universal stress proteins and lacks an N-terminal signal peptide, its presence in the cytosol after nutrient deprivation was expected [50, 63]. Fourth, a GFP-fusion protein (SP-GFP), which was appended with an N-terminal signal peptide from the Luke(2) lectin and expressed under a GAPDH promoter, localized to secretory vesicles of cysts but not to cyst walls (S5 Fig). Fifth, GFP alone expressed under the GAPDH promoter was homogenously distributed in the cytosol of cysts (S6 Fig).

### Luke(2), Leo, and Jonah(1) lectins all bound to microcrystalline cellulose, while binding of cyst wall lectins to chitin beads was variable

**Fig 8:**
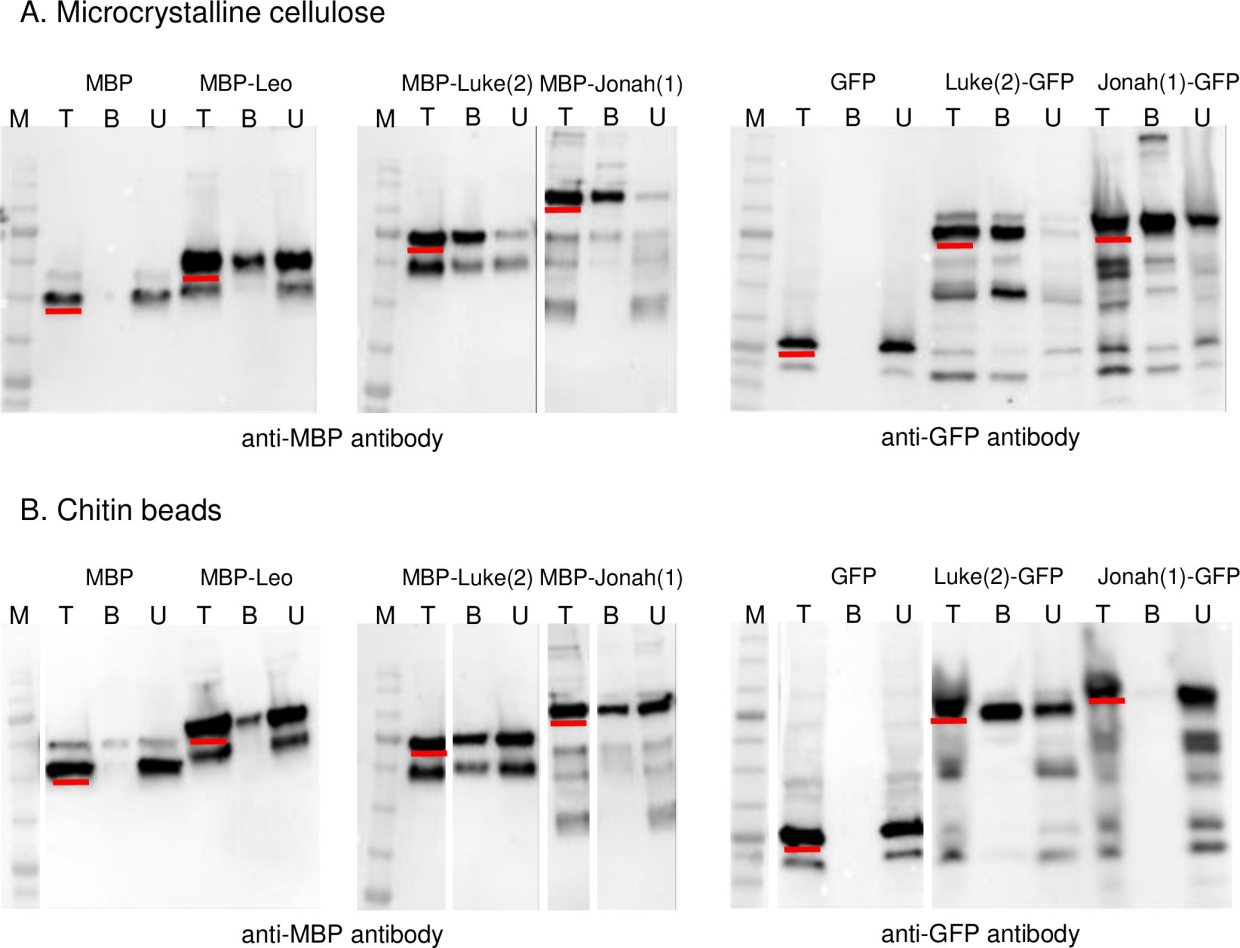
Western blots showed Luke(2), Leo, and Jonah(1) lectins fused to MBP or tagged with GFP bound well to microcrystalline cellulose, while binding to chitin beads was variable. MBP-lectin fusions and MBP alone, which were made as recombinant proteins in the periplasm of bacteria, were incubated with microcrystalline cellulose (A) or chitin beads (B). Total proteins (T), bound proteins (B), and unbound proteins (U), as well as molecular weight markers (M), were run on SDS-PAGE, transferred to PVDF membranes, and detected with an anti-MBP reagent. Full-length products in total fractions are underlined in red. MBP-Leo partially bound to microcrystalline cellulose and bound weakly, if at all to chitin. MBP-Luke(2) and MBP-Jonah(1) each bound more completely to cellulose than to chitin. MBP alone (negative control) did not bind to cellulose or chitin. Luke(2)-GFP, Jonah(1)-GFP, and GFP alone, each of which was expressed under the GAPDH promoter, were released from lysed trophozoites and incubated with microcrystalline cellulose or chitin beads. Luke(2)-GFP, which included some breakdown products, bound completely to cellulose and partially to chitin. Jonah(1)-GFP, which also included some breakdown products, bound partially to microcrystalline cellulose but not at all to chitin. GFP alone (negative control) did not bind to cellulose or chitin.

To test the binding of abundant cyst wall proteins to commercially available glycopolymers, we made MBP-cyst wall protein fusion-proteins in the periplasm (secretory compartment) of *E*. *coli* [42]. Previously, we used MBP-fusions to characterize carbohydrate-binding properties of cyst wall lectins of *Entamoeba*, *Giardia*, and *Toxoplasma* [64–66]. The targets were microcrystalline cellulose (used to characterize binding activities of GST-SlCBM49 from tomato cellulase) and chitin beads (used to characterize myc-tagged Jacob and Jessie lectins of *Entamoeba histolytica*) [33, 67]. Western blots with anti-MBP antibodies showed MBP-Luke(2) and MBP-Jonah(1) each bound to microcrystalline cellulose and somewhat less well to chitin beads (Fig 8). MBP-Leo bound less completely to microcrystalline cellulose and weakly at best to chitin beads. MBP alone (negative control) did not bind to microcrystalline cellulose or chitin beads. As a control, we incubated with Luke(2)-GFP, Jonah(1)-GFP, and GFP alone, each obtained from lysates of trophozoites expressing the tagged proteins under a GAPDH promoter with cellulose and chitin. Consistent with the MBP-fusions, Luke(2)-GFP and Jonah(1)-GFP bound to cellulose and Luke(2)-GFP bound to chitin, while GFP alone bound to neither cellulose nor chitin. The one discrepant finding was that Jonah(1)-GFP failed to bind to chitin, to which MBP-Jonah(1) bound (Fig 8).

These results showed abundant Luke(2), Leo, and Jonah(1) lectins each bound cellulose well, while binding to chitin was much more variable. The binding patterns of tagged Luke, Leo, and Jonah lectins to cellulose and chitin *in vitro*, however, were poor predictors for localization of these proteins in mature cyst walls.

### GFP-tagged lectins and glycopolymers were accessible in ectocyst layer but were inaccessible in the endocyst layer and ostioles of mature cyst walls

**Fig 9:**
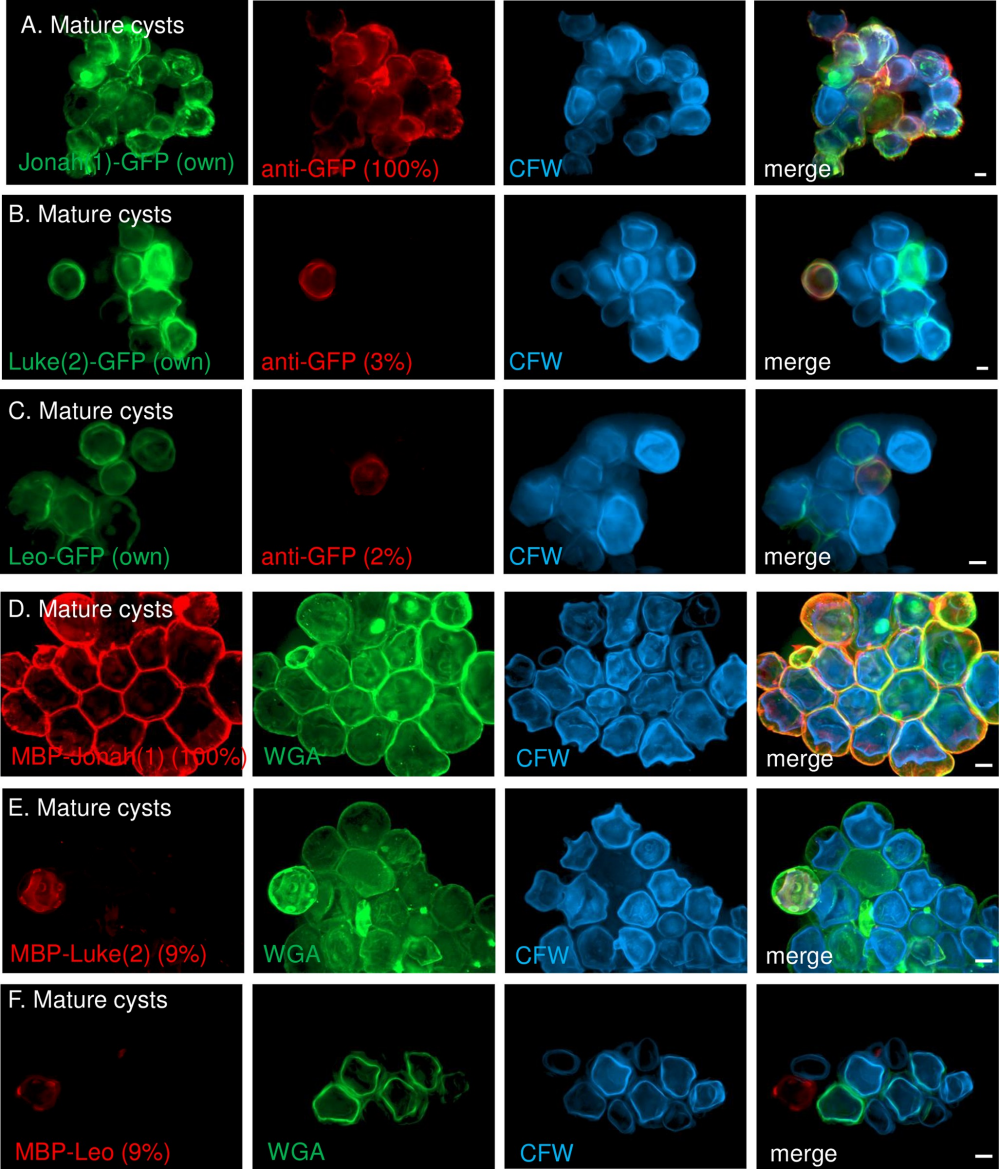
Widefield microscopy showed Jonah(1)-GFP and glycopolymers labeled by MBP-Jonah(1) were accessible in the ectocyst layer of mature cyst walls, while Luke(2)-GFP, Leo-GFP, and glycopolymers labeled by MBP-Luke(2) and MBP-Leo were inaccessible in the endocyst layer and ostioles. A. Nearly 100% of mature cysts expressing Jonah(1)-GFP (green) under its own promoter were labeled with anti-GFP antibodies (red). CFW (blue) labeled the endocyst layer of cyst walls. B. Just 3% of mature cysts expressing Luke(2)-GFP under its own promoter labeled with anti-GFP antibodies. C. Just 2% of mature cysts expressing Leo-GFP under its own promoter labeled with anti-GFP antibodies. D. MBP-Jonah(1) (red) labeled 100% of mature cysts, which were also labeled with WGA (green) and CFW. In contrast, MBP-Luke(2) (E) and MBP-Leo (F) each labeled 9% of mature cysts. While it was sometimes possible to distinguish the ectocyst and endocyst layers with widefield and DIC microscopy, it was not easy. Similarly, it was very difficult to identify ostioles with widefield microscopy and impossible with DIC. A to F. Scale bars are 5 μm. SIM of mature cysts labeled with MBP-lectin fusions are shown in S7 Fig.

To determine the accessibility of proteins in the ectocyst and endocyst layers and ostioles of mature cyst wall, we incubated organisms expressing GFP-tagged lectins under their own promoters with anti-GFP antibodies. Widefield microscopy showed that Jonah(1)-GFP was accessible in the endocyst layer of nearly 100% of mature cysts with a detectable Jonah(1)-GFP signal (Fig 9A). In contrast, anti-GFP antibodies showed Luke(2)-GFP and Leo-GFP were accessible in the endocyst layer and ostioles of 3 and 2%, respectively, of mature cysts with detectable GFP signals (Fig 9B and 9C).

To determine the accessibility of glycopolymers in two layers of mature cyst walls, we labeled cysts with MBP-lectin fusion-proteins. MBP-Jonah(1) bound to the ectocyst layer of 100% of mature cell walls (Fig 9D and S7 Fig), which was the same location as Jonah(1)-GFP expressed under either its own or the GAPDH promoter (Fig 7A and 7E). In contrast, MBP-Luke(2) and MBP-Leo probes each labeled the endocyst layer and ostioles of 9% mature cyst walls (Fig 9E and 9F and S7 Fig). Although these were the same places in mature cyst walls where Luke(2)-GFP and Leo-GFP localized under either their own promoters or the GAPDH promoter (Luke(2)-GFP) (Fig 7B, 7C and 7F), these results suggested that glycopolymers bound by MBP-Luke(2) and MBP-Leo in the endocyst layer and ostioles were, for the most part, inaccessible to external probes.

Finally, by rotating three-dimensional SIM reconstructions of organisms expressing Luke(2)-GFP or Leo-GFP or labeled with WGA, MBP-Luke(2), or MBP-Leo, we counted an average of 8.8 +/- 2.5 ostioles per mature cyst wall (24 cysts total). To our knowledge, this is the first estimate of the number of ostioles in *Acanthamoeba* cyst walls, because ostioles have not previously been visualized by light microscopy and are extremely difficult to count by TEM [20], unless dozens of serial sections are performed.

## Discussion

### Familiarity and novelty in *A*. *castellanii* cyst wall lectins

Although we expected Luke lectins with two or three CBM49s would be present in the cellulose-rich cyst wall, we could not have predicted the other abundant cyst wall proteins, because the 8-Cys domains of Leo lectins are unique to *Acanthamoebae* and the CAA domains of Jonah lectins were previously uncharacterized [33–35, 53–57]. While Luke lectins have two or three CBM49s, *D*. *discoideum* has dozens of proteins with a single CBM49 (S8 Fig). The Luke lectins bind cellulose and chitin, while the *D*. *discoideum* proteins with a single CBM49 bind cellulose [56]. Chitin-binding by DdCBM49 or SlCBM49 was not tested, because this glycopolymer is not present in *D*. *discoideum* and tomato walls. Demonstration that CBM49s of the Luke lectin also bind chitin fibrils is new, but is consistent with recent studies showing CBMs may bind more than one glycopolymer [55]. The metalloprotease fused to an N-terminal CBM49 of *A*. *castellanii* is absent in *D*. *discoideum*, while *D*. *discoideum* adds two CBM49s to a cysteine proteinase, which lacks these domains in *A*. *castellanii*. The CBM49 may act to localize the metalloproteases to the *A*. *castellanii* cyst wall, as is the case for the chitin-binding domain in *Entamoeba* Jessie lectins or glucan-binding domain in a *Toxoplasma* glucanase [64, 66, 67]. Alternatively, the CBM49 may suggest the metalloprotease cleaves glycopeptides rather than peptides. While the GH5 glycoside hydrolases of *A*. *castellanii* lack CBM49s, CBM49 is present at the C-terminus of GH9 glycoside hydrolases of *D*. *discoideum* and *S*. *lycopersicum* [33, 34].

Even though *A*. *castellanii* Leo lectins and *E*. *histolytica* Jacob lectins share no common ancestry, they have 8-Cys and 6-Cys lectin domains, respectively, often separated by low complexity sequences (S9 Fig) [67, 68]. *E*. *histolytica* low complexity sequences vary from strain to strain, contain cryptic sites for cysteine proteases, and are extensively decorated with *O*-phosphate-linked glycans [69]. We have not yet identified any Asn-linked or *O*-linked glycans on Leo lectins or any of the other cyst wall proteins, but we expect they will be there. *A*. *castellanii* and oomycetes (*Pyromyces* and *Neocallmistic*) each contain proteins with arrays of CAA domains, but the sequences of the CAAs are so different that it is likely that concatenation of domains occurred independently (S10 Fig) [53, 54]. Although *A*. *castellanii* is exposed to collagen in the extracellular matrix of the cornea, the protist lacks a homolog of the collagen-binding domain that is adjacent to the CAA domain in the *Bacillus anthracis* collagen-binding protein [57]. Concatenation of carbohydrate-binding domains in Luke, Leo, and Jonah(3) lectins, which has previously been shown in WGA, Jacob lectins of *E*. *histolytica*, and peritrophins of insects, most likely increases the avidity of the lectins for glycopolymers [67, 68, 70].

### Three distinct stages in the development of the *A*. *castellanii* cyst wall

While the boundaries between the three stages of the development of the cyst wall were somewhat arbitrary (based upon the times selected for examining cyst walls with SIM), each stage had several essential, distinguishing features. In the first stage, encysting organisms rapidly and in an explosive manner transformed from amoeboid trophozoites, which were full of vacuoles and have acanthopods, to immotile, rounded forms making glycopolymers and Jonah(1) lectins in dozens of distinct vesicles. Because vesicles labeled with WGA and GST-CBM49 did not overlap, it is likely that they contain different glycopolymers. Definitive identification of glycopolymers in vesicles of encysting organisms will depend upon localization of tagged cellulose and chitin synthases, each of which is encoded by a single gene in *A*. *castellanii* [1, 34–37]. Encysting *Entamoebae* also transform from amoeboid trophozoites to immotile, rounded forms making chitin, chitinase, and the Jacob lectin in distinct vesicles [45, 64]. Encysting *Giardia* also transform from flagellated forms with an adherence disc to an spherical, immotile forms making β-1,3-linked GalNAc glycopolymer and cyst wall proteins (CWP1 to CWP3) in distinct vesicles [65]. In contrast, no dramatic secretory event occurs in fungi or plants, which remodel their walls with growth, differentiation, or cell division, but never make their walls from scratch, with exception of the septum separating dividing cells [71, 72].

In second stage, the two most remarkable features of the primordial cyst wall were the distinct distributions of the GFP-tagged lectins and the sets of small, circular ostioles. Early on, Jonah(1)-GFP was homogenously distributed across the primordial wall, while Luke(2)-GFP outlined some ostioles. Later, Luke(2)-GFP spread across the primordial wall, while Leo-GFP appeared in patches, which were not specific to any structure. It was as if Leo-GFP was secreted onto the surface of second stage organisms but had not yet found the glycopolymer to which it binds. Because the ostioles labeled with the least specific external probe (CFW), it was not possible to determine whether ostioles are composed of cellulose, chitin, or another glycopolymer. Indeed CFW has been shown to bind numerous β-1,3 and β-1,4 polysaccharides, including cellulose, chitin, mixed linkage glucans, and galactoglucomannan [44, 46, 47]. The presence of the internal probe Luke(2)-GFP in the small ostioles did not settle this problem, as Luke(2)-GFP extracted from lysed trophozoites bound to both microcrystalline cellulose and chitin beads. As ostioles labeled with CFW before they contained Luke(2)-GFP, it is likely that glycopolymers are the drivers behind the circular or ring-like structures. How the small ostioles simultaneously appear in an even distribution across the surface of the primordial cyst wall and synchronously develop into conical structures is of great interest but is beyond the scope of the present study. Because *A*. *castellanii* has almost nine ostioles but uses just one for the excysting trophozoite to escape the cyst wall, it is likely that ostioles serve other functions such as holding layers of the cyst wall together and/or exchanging nutrients or waste products with the environment.

In the third stage, the Jonah(1) lectin and the glycopolymer that it binds remained in the ectocyst layer, while Luke(2) and Leo lectins and the glycopolymers that they bind move to the endocyst layer and ostioles. In the same way, the outer primary layer of plant cells forms before the inner secondary layer [72]. The distinct distributions of the three GFP-tagged lectins in the primordial, third stage, and mature cyst walls strongly suggests each lectin binds to different glycopolymers (e.g. cellulose versus chitin), glycopolymers modified in different ways (e.g. unmodified chitin versus deacetylated chitosan), and/or glycopolymers with different microfibrillar structures (e.g. microcrystalline versus amorphous cellulose). In support of this idea, the lectins had the same localization in mature cyst walls when expressed as an internal probe with a GFP-tag under its own or under a constitutive GAPDH promoter or when applied externally as an MBP-fusion. There may also be protein-protein interactions and/or lectin-glycoprotein interactions, which determine the localization of cyst wall lectins in the *A*. *castellanii* cyst wall. As an example of protein-protein interactions, an *E*. *histolytica* Jessie lectin has a chitin-binding domain and a self-agglutinating “daub” domain, which makes cyst walls impermeable to small probes such as phalloidin [64]. As an example of lectin-glycan interactions, the Gal/GalNAc lectin on the plasma membrane of *Entamoebae* binds to glycans on Jacob lectins, which, in turn, bind to chitin fibrils in the cyst wall [45].

Anti-GFP antibodies and MBP-lectin fusions showed Jonah(1) lectin and glycopolymers to which it binds are accessible in the ectocyst layer of mature cyst walls, while Luke(2) and Leo lectins and the glycopolymers to which they bind are, for the most part, inaccessible in the endocyst layer and ostioles. Jonah(1) lectin, which is unique, abundant, accessible, and conserved across many strains, therefore, is an excellent target for diagnostic antibody to *A*. *castellanii* cysts. Diagnostic antibodies bind to abundant cyst wall protein 1 of *Giardia* and Jacob2 lectin of *Entamoeba* [73, 74]. In contrast, Luke(2) and Leo lectins are inaccessible and so not good targets for diagnostic antibodies.

### Caveats

While abundant Luke(2) and Luke(3) lectins with two or three CBM49s, respectively, localized to the endocyst layer and ostioles of mature cyst walls, we have no evidence that other less abundant Luke lectins localize in the same place. In the same way, we do not know whether other Leo lectins localize to the endocyst layer and ostioles, or other Jonah lectins localize to the ectocyst layer. The large number of genes encoding Luke, Leo, and Jonah lectins may simply be necessary to increase the quantity of proteins coating glycopolymers in the cyst wall. Alternatively, there may be differences in timing and localization of proteins within the same family, based upon a TKH-rich spacer in Leo lectins or transmembrane helices in Jonah(3) lectins with three CAA domains (S2 Fig). Finally, other candidate cyst wall proteins, which are abundant but present at lower copy numbers (e.g. laccase or ferritin-domain protein) and so not tested here, may have important functions in the cyst wall.

While localization of abundant lectins was highly reproducible throughout cyst wall development, identification of the glycopolymers to which they bound was much more difficult. First, while each of the lectins bound well to microcrystalline cellulose, the lectins also bound to varying degrees to chitin beads. Second, the N-terminal CBM49 of a Luke(2) lectin fused to GST bound to the ectocyst layer of mature cyst walls, while N- and C-terminal CBM49s of the same Luke(2) lectin fused to MBP bound to the endocyst layer and ostioles. Third, WGA, bound to the ostioles and endocyst layer, the ectocyst layer, or all three structures of mature cyst walls, depending upon the experiment. Fourth and finally, experiments with anti-GFP antibodies and MBP-lectin fusions showed that proteins and glycopolymers in the endocyst layer and ostioles of mature cyst walls are, for the most part, inaccessible to external probes. We, therefore, cannot make any conclusions at this time as to the locations of cellulose and chitin in developing and mature cyst walls. In particular, we do not think it is a simple as cellulose in the ectocyst layer and chitin in the endocyst layer, as suggested by binding of GST-CBM49 and WGA in Fig 2. However, localization of cellulose and/or chitin in vesicles of encysting organisms and in cyst walls is a solvable problem with 1) more specific probes for each glycopolymer, 2) GFP-tags for cellulose and chitin synthases that are each encoded by single genes, 3) protease or chemical treatments to expose glycopolymers to external probes, cellulases, and chitinases, and/or 4) inhibition of chitin and cellulose synthases with pharmacological agents or silencing RNAs. Indeed other investigators have explored the possibility of cellulose synthase inhibitors or cellulases as therapeutics versus *A*. *castellanii* cysts [75–78].

## Supporting information

S1 Fig Constructs made to localize Ac cyst wall proteins and to determine their binding to microcrystalline cellulose and chitin beads. A. An abundant Luke(2) lectin with two CBM49s was used to make GFP-, GST-, and MBP-fusions. An abundant Luke(3) lectin with three CBM49s was used to make a GFP-fusion protein. B. An abundant Leo lectin was made into GFP- and MBP-fusions. C. An abundant Jonah(1) lectin with a single CAA domain was made into GFP- and MBP-fusions. D. Vectors for expressing GFP-fusions in transfected *A*. *castellanii* under its own promoter or under the GAPDH promoter contained a neomycin resistance gene under a TATA-binding protein promoter [40, 41]. Primers for making constructs are listed in S1 Excel file.
(PPTX)

S2 Fig Sequences of candidate cyst wall proteins, which differ in at least one essential property from Luke(2), Leo, and Jonah(1) lectins that were used for localization and binding studies. A Luke(3) lectin is comprised of an N-terminal signal peptide (purple) and three CBM49s separated by short Ser- and Pro-rich spacers (light blue). The CBM49s contain conserved Trp (red Ws) present in the abundant Luke(2) lectin (Fig 3). A Leo(TKH) lectin is comprised of a signal peptide, two domains containing eight Cys residues each (red Cs), and a long Thr-, Lys-, and His-rich spacer (brown). A Jonah(3) lectin is comprised of three CAA domains (green), hydrophobic regions (tan), and short Ser- and Pro-rich spacers (light blue).
(PDF)

S3 Fig RT-PCR shows mRNAs of abundant Luke(2), Leo, and Jonah(1) lectins, as well as those of cellulose synthase, are encystation-specific. DNA and total RNA were extracted from trophozoites and organisms encysting for one to three days. RT-PCRs were performed with primers specific for segments of each cyst wall protein mRNA, as well as primers specific for segments of mRNAs for GAPDH and cellulose synthase (S1 Excel file). PCR with DNA was used as a positive control, while omission of reverse-transcriptase (-RT) was used as a negative control. Messenger RNAs encoding cyst wall proteins and cellulose synthase were absent or nearly absent in trophozoites but were easily detectable in encysting organisms. In contrast, mRNAs for GAPDH were expressed by both trophozoites and encysting organisms [41].
(PDF)

S4 Fig Western blots with rabbit antibodies to peptides of Jonah(1) and Leo lectins show each lectin is absent in trophozoites but is easily detected in mature cysts. A. Coomassie blue stain of proteins of lysed trophozoites and cysts, as well as molecular weight standards (M). B. Western blotting showed rabbit antibodies to a 50-amino acid peptide of an abundant Jonah(1) lectin (underlined in Fig 3) bound to a cyst protein of the predicted size (red underline) and to an MBP-Jonah(1) fusion-protein made in the periplasm of bacteria. The antibody also bound to degradation products of Jonah(1) lectin. In contrast, the anti-Jonah(1) antibody did not bind to either trophozoites or MBP alone (negative controls). C. Rabbit antibodies to a 16-amino acid peptide of an abundant Leo lectin also bound to cyst proteins and to an MBP-Leo fusion but not to trophozoite proteins or to MBP alone. In addition to Leo of the predicted size (red underline), anti-Leo antibodies bound to a higher molecular weight form, which may be a dimer. These results confirmed encystation-specific expression of Jonah(1) and Leo lectins (Figs 4 to 6). None of the rabbit anti-peptide antibodies reacted with native proteins, and so they were not useful for labeling cyst walls for widefield microscopy or SIM.
(PDF)

S5 Fig SIM showed control GFP constructs localize to the cytosol (CSP21-GFP) and secretory vesicles (GFP with an N-terminal signal peptide, SP-GFP) of mature cysts. A. The 21-kDa cyst-specific protein (CSP21) fused to GFP was absent in trophozoites but formed punctate structures in the cytosol of cysts [28]. B. GFP with an N-terminal signal peptide from Luke(2) lectin and expressed under a GAPDH promoter localized to secretory vesicles of mature cysts [41]. These controls make it unlikely that localizations of candidate cyst wall proteins-tagged with GFP in mature cysts were artifacts (Fig 7). Scale bars are 2 μm.
(PDF)

S6 Fig Widefield and DIC microscopy showed Luke(2) and Jonah(1) lectins tagged with GFP and expressed under a constitutive GAPDH promoter localized to secretory vesicles of trophozoites, while GFP alone expressed under the GAPDH promoter localized to the cytosol of trophozoites and cysts. A. Luke(2)-GFP (green) under the GAPDH promoter localized to small vesicles, which were distinct from larger vacuoles (white arrows) in a trophozoite that retained acanthopods on its surface (black arrows). B. Jonah(1)-GFP also under the GAPDH promoter localized to small vesicles that were distinct from larger vacuoles. In contrast, GFP alone, which was also expressed under the GAPDH promoter, diffusely labeled the cytosol of trophozoites and cysts. A, B. Scale bars are 5 μm.
(PDF)

S7 Fig SIM showed glycopolymers bound by MBP-Jonah(1) were accessible in the ectocyst layer of mature cyst walls, while glycopolymers bound by MBP-Luke(2) and MBP-Leo were mostly inaccessible in the endocyst layer and ostioles. Although MBP-Jonah(1) labeled the ectocyst layer of nearly 100% of mature cysts (A), MBP-Luke(2) (B) and MBP-Leo (C) each labeled the endocyst layer and ostioles of 9% of mature cysts. A to C. Scale bars are 2 μm.
(PDF)

S8 Fig Contrasting uses of CBM49 by *A. castellanii* and *D. discoideum*. CBM49, which was first shown to be a cellulose-binding domain at the C-terminus of tomato cellulase [34, 35], is repeated two or three times in Luke lectins of *A*. *castellanii* and is also present at the N-terminus of a metalloprotease. In contrast, CBM49 is present in a single copy in the majority of *D*. *discoideum* proteins and as three copies in rare proteins [54, 56]. CBM49 is also present in two copies in a *D*. *discoideum* cysteine protease and as a single copy in a GH5 glycoside hydrolase.
(PDF)

S9 Fig *A. castellanii* Leo lectins and *E. histolytica* Jacob lectins have common structures, even though they share no common ancestry (convergent evolution). Abundant cyst wall proteins of *A*. *castellanii* (Leo lectins) and *E*. *histolytica* (Jacob lectins) have unique 8-Cys or 6-Cys domains, respectively, that bind cellulose or chitin [67, 68]. In each protist, some of the lectins lack spacers, while others have spacers rich in Thr, Lys, and His (*A*. *castellanii*) or Ser and Thr (*E*. *histolytica*).
(PDF)

S10 Fig Contrasting use of choice of anchor A (CAA) domains in *A. castellanii*, oomycetes, and bacteria. Jonah lectins, which are abundant in cyst walls of *A*. *castellanii*, have one or three CAA domains. The former are preceded by Thr-, Lys-, and Cys-rich sequences (gray), while the latter are separated by Ser- and Pro-rich spacers (blue) and hydrophobic domains (tan). Predicted proteins of oomycetes (*Pyromyces* or *Neocallimastix*) have three to five CAA domains, while the spore coat protein of *Bacilllus* has a single CAA domain attached to a collagen-binding domain, which is absent in *A*. *castellanii* [53, 57].
(PDF)

S1 Excel file Primers used for RT-PCR and construction of GFP- and MBP-fusions. (XLSX)

S2 Excel file This file lists the most abundant proteins identified by mass spectrometry of cyst walls purified on the Percoll gradient and retained on a membrane containing 8-μm pores. Proteins with <7 unique peptides have been removed, because they are dominated by cytosolic contaminants. Luke, Leo, and Jonah lectins have been highlighted in orange. Other candidate cyst wall proteins are marked in yellow.
(XLSX)

S3 Excel file Complete list of proteins identified by mass spectrometry of cyst walls. This list includes a preparation that was heavily contaminated with cytosolic proteins, because the Percoll gradient and porous membrane were omitted during their purification. Only proteins with at least two unique peptides are included.
(XLSX)
