# Supplementary material for: The most abundant cyst wall proteins of Acanthamoeba castellanii are lectins that bind cellulose and localize to distinct structures in developing and mature cyst walls
Source: PLoS Negl Trop Dis. 2019 May 16;13(5):e0007352. doi: 10.1371/journal.pntd.0007352 (PMC6541295; doi:10.1371/journal.pntd.0007352)
Supplement: S1 Fig — A. An abundant Luke(2) lectin with two CBM49s was used to make GFP-, GST-, and MBP-fusions. An abundant Luke(3) lectin with three CBM49s was used to make a GFP-fusion protein. B. An abundant Leo lectin was made into GFP- and MBP-fusions. C. An abundant Jonah(1) lectin with a single CAA domain was made into GFP- and MBP-fusions. D. Vectors for expressing GFP-fusions in transfected A. castellanii under its own promoter or under the GAPDH promoter contained a neomycin resistance gene under a TATA-binding protein promoter [40, 41]. Primers for making constructs are listed in S1 Excel file. (PPTX) [file pntd.0007352.s001.pptx]

## Slide 1
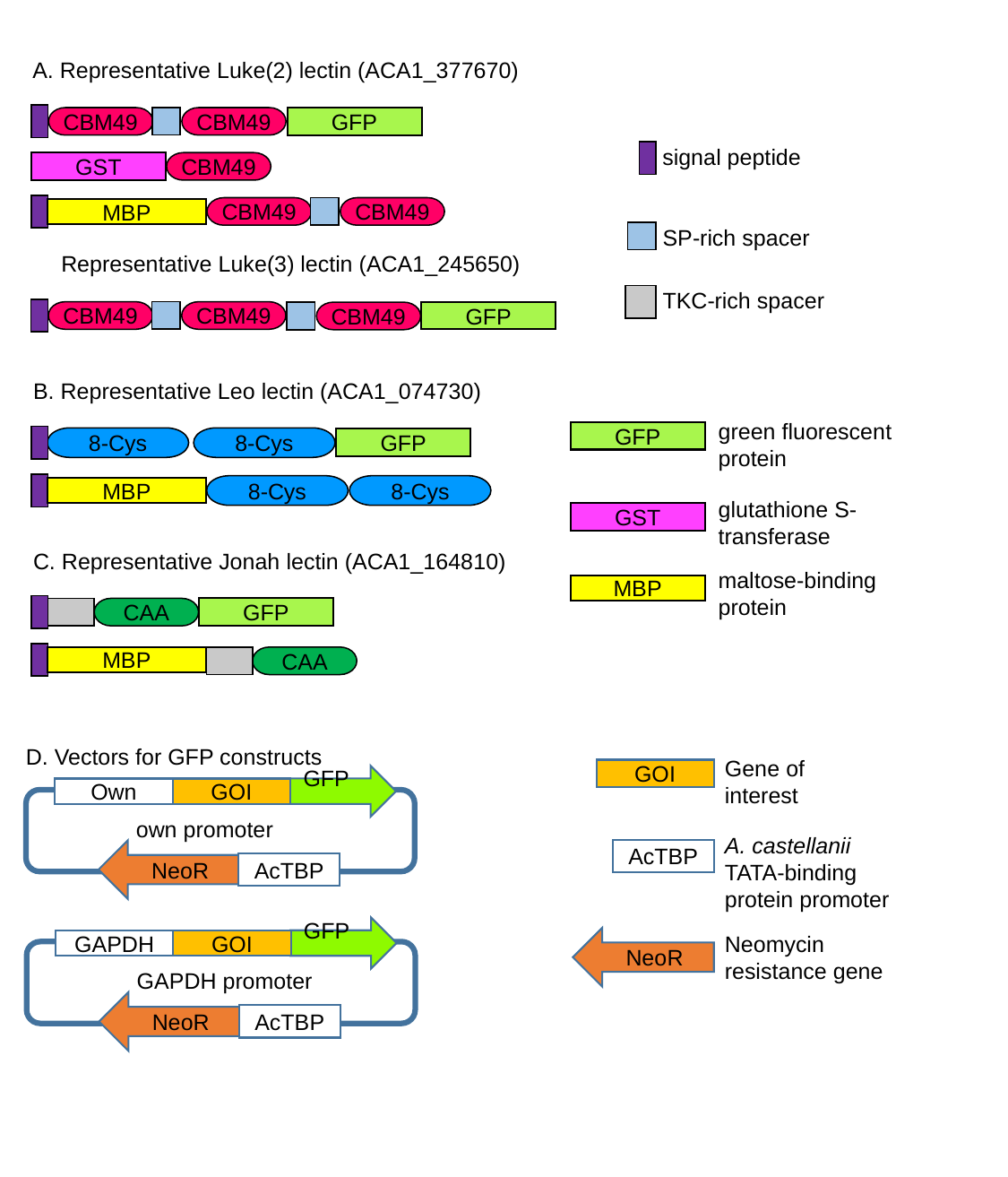

A. Representative Luke(2) lectin (ACA1_377670)
CBM49
CBM49
GFP
signal peptide
GST
CBM49
CBM49
CBM49
MBP
SP-rich spacer
Representative Luke(3) lectin (ACA1_245650)
TKC-rich spacer
CBM49
CBM49
GFP
CBM49
B. Representative Leo lectin (ACA1_074730)
green fluorescent protein
GFP
8-Cys
8-Cys
GFP
8-Cys
8-Cys
MBP
glutathione S-transferase
GST
C. Representative Jonah lectin (ACA1_164810)
maltose-binding protein
MBP
CAA
GFP
MBP
CAA
D. Vectors for GFP constructs
Gene of interest
GOI
GFP
Own
GOI
own promoter
A. castellanii TATA-binding protein promoter
AcTBP
 NeoR
AcTBP
GFP
GAPDH
GOI
Neomycin resistance gene
 NeoR
GAPDH promoter
 NeoR
AcTBP
