# Supplementary material for: The most abundant cyst wall proteins of Acanthamoeba castellanii are lectins that bind cellulose and localize to distinct structures in developing and mature cyst walls
Source: PLoS Negl Trop Dis. 2019 May 16;13(5):e0007352. doi: 10.1371/journal.pntd.0007352 (PMC6541295; doi:10.1371/journal.pntd.0007352)
Supplement: S2 Fig — A Luke(3) lectin is comprised of an N-terminal signal peptide (purple) and three CBM49s separated by short Ser- and Pro-rich spacers (light blue). The CBM49s contain conserved Trp (red Ws) present in the abundant Luke(2) lectin (Fig 3). A Leo(TKH) lectin is comprised of a signal peptide, two domains containing eight Cys residues each (red Cs), and a long Thr-, Lys-, and His-rich spacer (brown). A Jonah(3) lectin is comprised of three CAA domains (green), hydrophobic regions (tan), and short Ser- and Pro-rich spacers (light blue). (PDF) [file pntd.0007352.s002.pdf]

Luke(3) lectin (ACA1_245650)

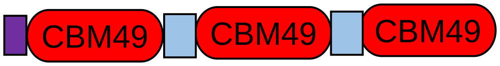

MKQQHQLVLLVFAALFCLTAA

DCAVRIEQSLGSAWNGAGGVPMSQWNARIVSSGSETVTALQVAFGAPTTAIDQVWGLEPVAGASRVY CBM49

DLPDYVILNGGLASGQAFNWGYIWESSAQAPLSVASVQC

SGGASPTAAPSASSSPVTAPSASSSPVTAPSASP

TIVASPAPCQLSVSQTTRPSSAGGSWTEGDFFFQIYDLTLLNSGSRPVSSAVIAIDTTSNQQQVITQFWN CBM49

LERQSATSDLFNVRNPGGNIEVGATLGAGYIVRTPLSAGSQVPPTTRLVSVNC

VGGPSPTIVATPQPSASPTIVASPQPSASPAAG

CNAAVSIVARSAAAGGSWTTGPNQFFQIFDITITNTGQRPLNGGVLTFGLPVAGSTITQWWELNRQGNT CBM49

NVFNVAFNFGPLLVGASQGAGIVVQTSSPSQALPSAVLSNLACAA

Leo(TKH) lectin (ACA1_374130)

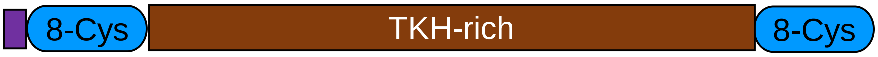

MTPATKLFLVLALGLVLVATAMA DDKKKHDSKGP

CKGGYYPDRKGCCPAIIKGKAYYRDGSKCYPKDVGCGVYYADGHGYYPDEKGNYAKDGEECPKERKCHERCIRPEHHEKS

TTTEECPKETTSTRRETTTTERRTTTPKRTTTTTEECPKETTTTKKHTTTTEDCPKETTTTKKHTTTTEECPKETTSTKR

HTTTTEECPKETTSTRKHSTSTKKHTTTTEECPKETTSTKKHYTTSTEECPKETTTTKKHTTTTEECPKETTTTKKHYTT

STTEECPKETTTTKKHYTTSTKKHHTTSTEECPKETTSTRKETTSTKKHHTTSTTEECPKETTSTRKETTSTRRETTSTK

KHTTTTEECRKTTTEKPCDDDHDKDNK

CPGGQWKDGKGCCPRKIGHQWYYRDAHGCYPVERDCGVFYADGKGYYPDENGQYAKAGEYCPEYRKCKPECDGGYGHY

Jonah(3) lectin (ACA1_157320)

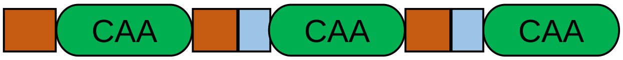

MSRSRHRVSAPFWGMLLLFSLAALACTCPVAQRSAEPVGLSVGLGVGQSLALALGRGLRFGFGFYSALCFGFRIGQQAAVAFCIR

VGFAGRQQVPFAFGIGLSLALAFCVGFGFGFRFRFGVGVAEQVGLAVSVGFGFAEQVSLALCFGIGVGIGLAIAHGFTVGIGLAI

AHGRNCTTALFNGAQSFDTFLFGSATIQNGQFGGRVAISGNANLTQFGIANALPCTNADARNFNLIVNGNLTSVNGVACGSALVT

GTVVSAPNFRQEAQGAGVVSGSASMAAVDFDQSFSSLLFTNQQICSLTCKQAAITGSGAVTFDFDNTTNVYCLNAADASRATSFR CAA

FTNFPVDFAGALAINILGSAGETVTLSNAATNLGNVKPTQIVWNVCNGLNVRLSAFTLFGSLLAPASSINITNAQQSLLR

ILLPLGLAFAHPVGLPIGLPLGLGLRFAFGCRLGVTFTLGLCHVITVGFRFALGVSLGGCFALGFGFTLCRRFGFSLGLCHVITV

GIPVRFALGGCVGLCFAFCGRFGFGFGIAGRQRVSLGFTFCGCLCLTLGLTLCGCLGFSVARRQRLGLALSGRFAIGFCFALCGR

FGFALRCCLGLGLARRHA

SASPAASASASPSASASPSAAASPTASPPIVGSPTPQPPII

GGNCSSPIKALDGAADFDTFVLGQGDALTARNGQFGGRVAVNGNARLSSFGMGAELVCAANDSSAFNLIVQGNLSATNGEIFCGS

ALVNGSIQSAPSLRQINQGANVTQGDLLTLSTLDFTESADNLTRINRQLCEAFADNCTQAEIDAFGIVSLRWDNVTNAGRAQVFC CAA

LNSSLIANATFFEFAGFPANFSGAIVVNILGSEDVRFANAAISAGNLNATQVLWNVCNATRVDIFAFQLFGSLLAPL

SNVSLINAQQIGTTVALNFTGSSFQKLLAPFIGEICLPDHRRHAHPVGHGLALTRRITLCGRLGIAVALCRGLAIGFALR

VSVGLALCLALGLALCLALGLALCLALGLALCRRLSLGFALRIAFGIALRIAVGVALA

SPSISASPSLSASPTASLSPSISASPSAVSPTLSPSVSPTHPSLSGSPSVSRSPSVSPTLSPSVSPTQSASPSISGSPSS

ASPTQSASPSVSPTASASPSPPSNNVVG

AGNCTNALFGAAQAFDLFVLGTGDQNVTTGLNVVNGQFAGRVAVNSGARLNDFGIGNSLNCTAANATNFNFIVQNDLNATNGELF

CGSALVGGNVTNPLTFRQVNEGANLVSGVDVSISGIDFASATSFLTGVNQAVCALNCTNVTLGQANELRFTGAAGANSTQIFCVR CAA

SADLSRAASIVFDFAANFTAPIVVNIQGSPNDTTASFRSAAIQRGRLRSEQLLWNVCDSLDVTLSQFQLFGSLLAPNSSLTISNA

EQEGITVVSTLSGQGFQKRLAPFNGFFCQPL
