# Supplementary material for: The most abundant cyst wall proteins of Acanthamoeba castellanii are lectins that bind cellulose and localize to distinct structures in developing and mature cyst walls
Source: PLoS Negl Trop Dis. 2019 May 16;13(5):e0007352. doi: 10.1371/journal.pntd.0007352 (PMC6541295; doi:10.1371/journal.pntd.0007352)
Supplement: S3 Fig — DNA and total RNA were extracted from trophozoites and organisms encysting for one to three days. RT-PCRs were performed with primers specific for segments of each cyst wall protein mRNA, as well as primers specific for segments of mRNAs for GAPDH and cellulose synthase (S1 Excel file). PCR with DNA was used as a positive control, while omission of reverse-transcriptase (-RT) was used as a negative control. Messenger RNAs encoding cyst wall proteins and cellulose synthase were absent or nearly absent in trophozoites but were easily detectable in encysting organisms. In contrast, mRNAs for GAPDH were expressed by both trophozoites and encysting organisms [41]. (PDF) [file pntd.0007352.s003.pdf]

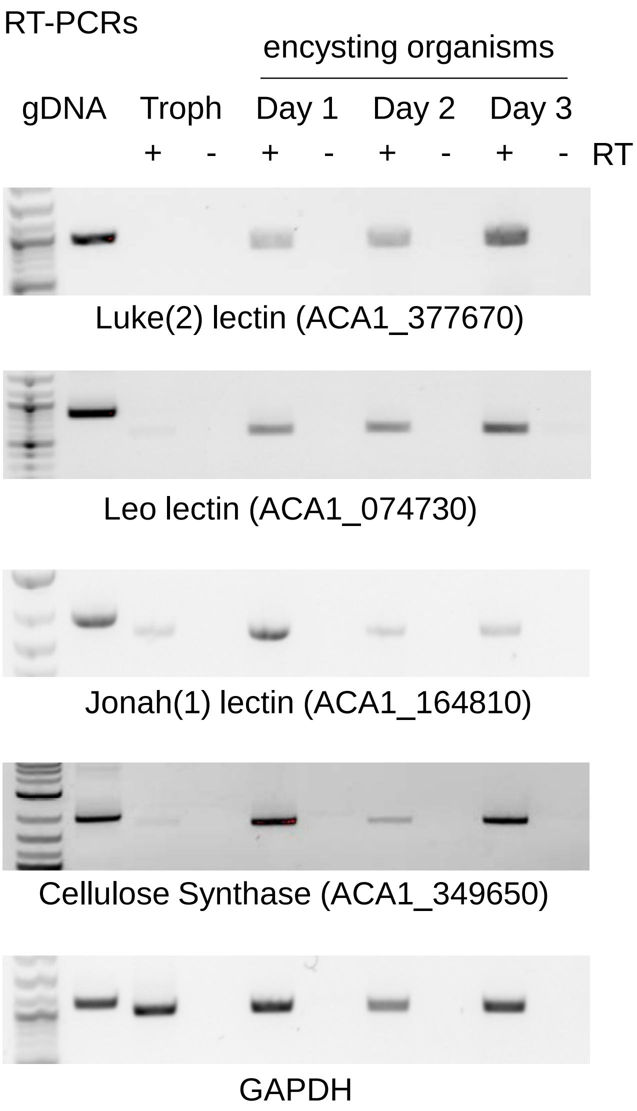
RT-PCRs
encysting organisms
gDNA
Troph
Day 1
Day 2
Day 3
+ - + - + - + - RT
Luke(2) lectin (ACA1_377670)
Leo lectin (ACA1_074730)
Jonah(1) lectin (ACA1_164810)
Cellulose Synthase (ACA1_349650)
GAPDH
