# Supplementary figures and images for: The most abundant cyst wall proteins of Acanthamoeba castellanii are lectins that bind cellulose and localize to distinct structures in developing and mature cyst walls
Source: PLoS Negl Trop Dis. 2019 May 16;13(5):e0007352. doi: 10.1371/journal.pntd.0007352 (PMC6541295; doi:10.1371/journal.pntd.0007352)

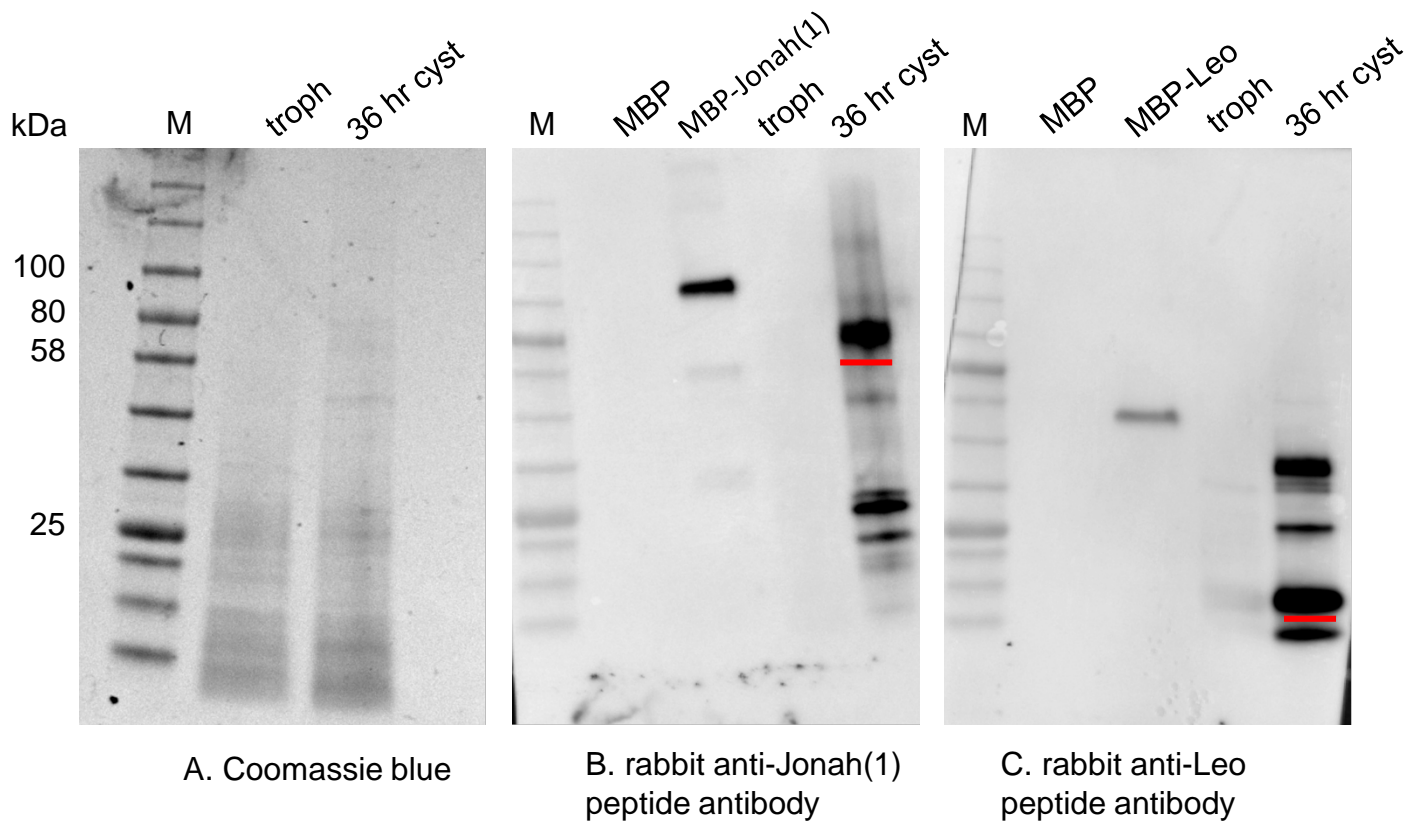

S4 Fig

Supplement: S4 Fig — A. Coomassie blue stain of proteins of lysed trophozoites and cysts, as well as molecular weight standards (M). B. Western blotting showed rabbit antibodies to a 50-amino acid peptide of an abundant Jonah(1) lectin (underlined in Fig 3) bound to a cyst protein of the predicted size (red underline) and to an MBP-Jonah(1) fusion-protein made in the periplasm of bacteria. The antibody also bound to degradation products of Jonah(1) lectin. In contrast, the anti-Jonah(1) antibody did not bind to either trophozoites or MBP alone (negative controls). C. Rabbit antibodies to a 16-amino acid peptide of an abundant Leo lectin also bound to cyst proteins and to an MBP-Leo fusion but not to trophozoite proteins or to MBP alone. In addition to Leo of the predicted size (red underline), anti-Leo antibodies bound to a higher molecular weight form, which may be a dimer. These results confirmed encystation-specific expression of Jonah(1) and Leo lectins (Figs 4 to 6). None of the rabbit anti-peptide antibodies reacted with native proteins, and so they were not useful for labeling cyst walls for widefield microscopy or SIM. (PDF) [file pntd.0007352.s004.pdf]

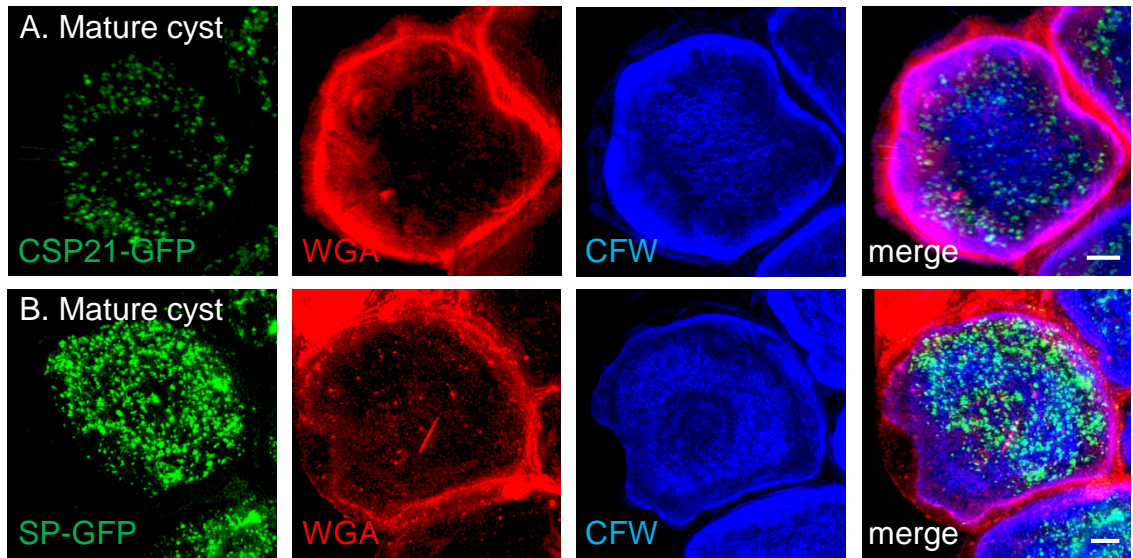

Supplement: S5 Fig — A. The 21-kDa cyst-specific protein (CSP21) fused to GFP was absent in trophozoites but formed punctate structures in the cytosol of cysts [28]. B. GFP with an N-terminal signal peptide from Luke(2) lectin and expressed under a GAPDH promoter localized to secretory vesicles of mature cysts [41]. These controls make it unlikely that localizations of candidate cyst wall proteins-tagged with GFP in mature cysts were artifacts (Fig 7). Scale bars are 2 μm. (PDF) [file pntd.0007352.s005.pdf]

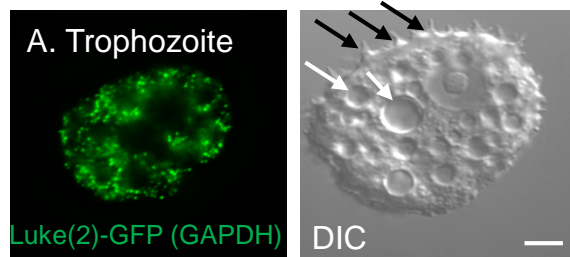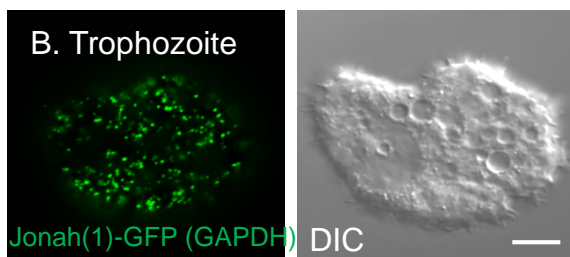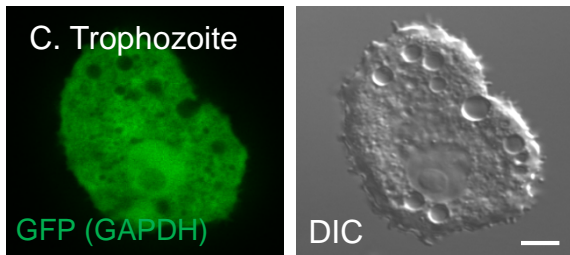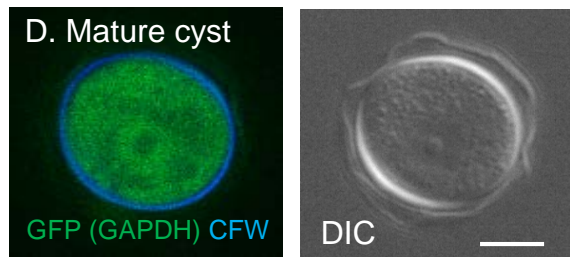

Supplement: S6 Fig — A. Luke(2)-GFP (green) under the GAPDH promoter localized to small vesicles, which were distinct from larger vacuoles (white arrows) in a trophozoite that retained acanthopods on its surface (black arrows). B. Jonah(1)-GFP also under the GAPDH promoter localized to small vesicles that were distinct from larger vacuoles. In contrast, GFP alone, which was also expressed under the GAPDH promoter, diffusely labeled the cytosol of trophozoites and cysts. A, B. Scale bars are 5 μm. (PDF) [file pntd.0007352.s006.pdf]

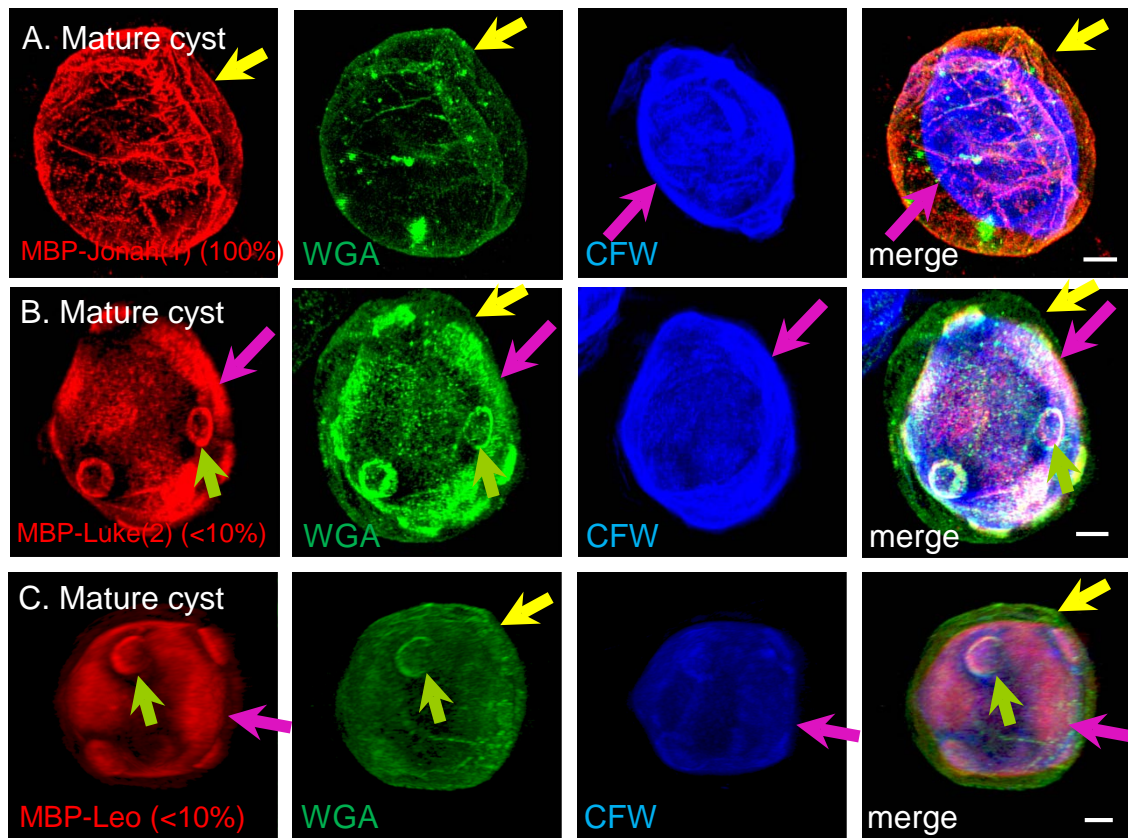

Supplement: S7 Fig — Although MBP-Jonah(1) labeled the ectocyst layer of nearly 100% of mature cysts (A), MBP-Luke(2) (B) and MBP-Leo (C) each labeled the endocyst layer and ostioles of 9% of mature cysts. A to C. Scale bars are 2 μm. (PDF) [file pntd.0007352.s007.pdf]

*A. castellanii* Luke lectins

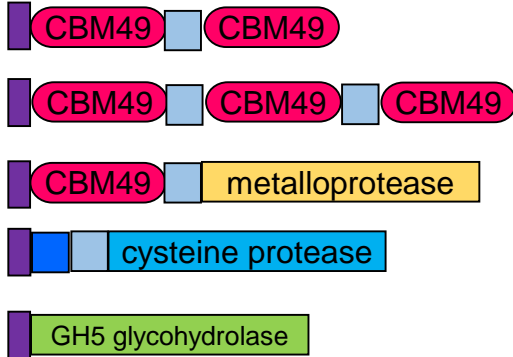

*D. discoideum* CBM49 proteins

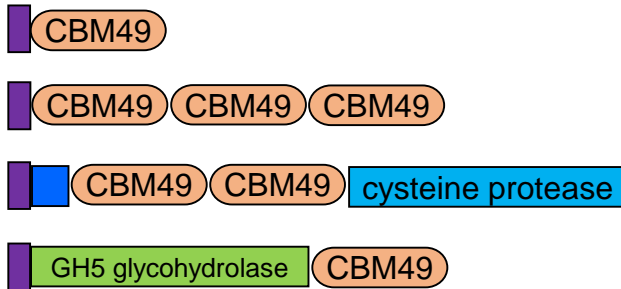

*Solanum lycopersicum* (tomato)

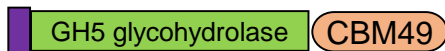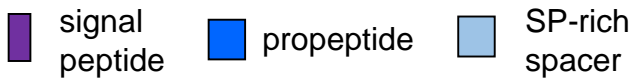

Supplement: S8 Fig — CBM49, which was first shown to be a cellulose-binding domain at the C-terminus of tomato cellulase [34, 35], is repeated two or three times in Luke lectins of A. castellanii and is also present at the N-terminus of a metalloprotease. In contrast, CBM49 is present in a single copy in the majority of D. discoideum proteins and as three copies in rare proteins [54, 56]. CBM49 is also present in two copies in a D. discoideum cysteine protease and as a single copy in a GH5 glycoside hydrolase. (PDF) [file pntd.0007352.s008.pdf]

*A. castellanii* Leo lectins

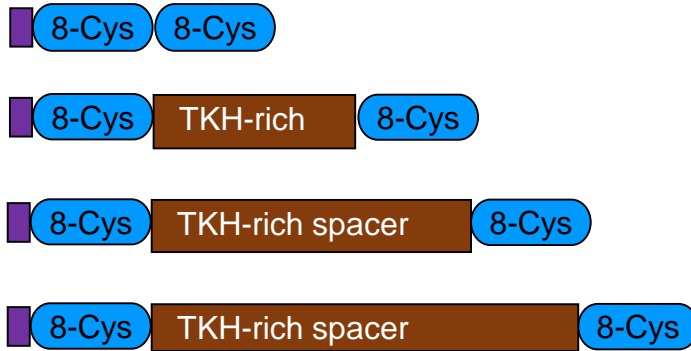

*E. histolytica* Jacob lectins

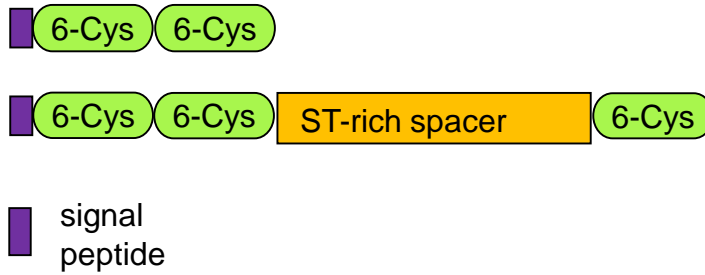

Supplement: S9 Fig — Abundant cyst wall proteins of A. castellanii (Leo lectins) and E. histolytica (Jacob lectins) have unique 8-Cys or 6-Cys domains, respectively, that bind cellulose or chitin [67, 68]. In each protist, some of the lectins lack spacers, while others have spacers rich in Thr, Lys, and His (A. castellanii) or Ser and Thr (E. histolytica). (PDF) [file pntd.0007352.s009.pdf]
