# Supplementary material for: The most abundant cyst wall proteins of Acanthamoeba castellanii are lectins that bind cellulose and localize to distinct structures in developing and mature cyst walls
Source: PLoS Negl Trop Dis. 2019 May 16;13(5):e0007352. doi: 10.1371/journal.pntd.0007352 (PMC6541295; doi:10.1371/journal.pntd.0007352)
Supplement: S10 Fig — Jonah lectins, which are abundant in cyst walls of A. castellanii, have one or three CAA domains. The former are preceded by Thr-, Lys-, and Cys-rich sequences (gray), while the latter are separated by Ser- and Pro-rich spacers (blue) and hydrophobic domains (tan). Predicted proteins of oomycetes (Pyromyces or Neocallimastix) have three to five CAA domains, while the spore coat protein of Bacilllus has a single CAA domain attached to a collagen-binding domain, which is absent in A. castellanii [53, 57]. (PDF) [file pntd.0007352.s010.pdf]

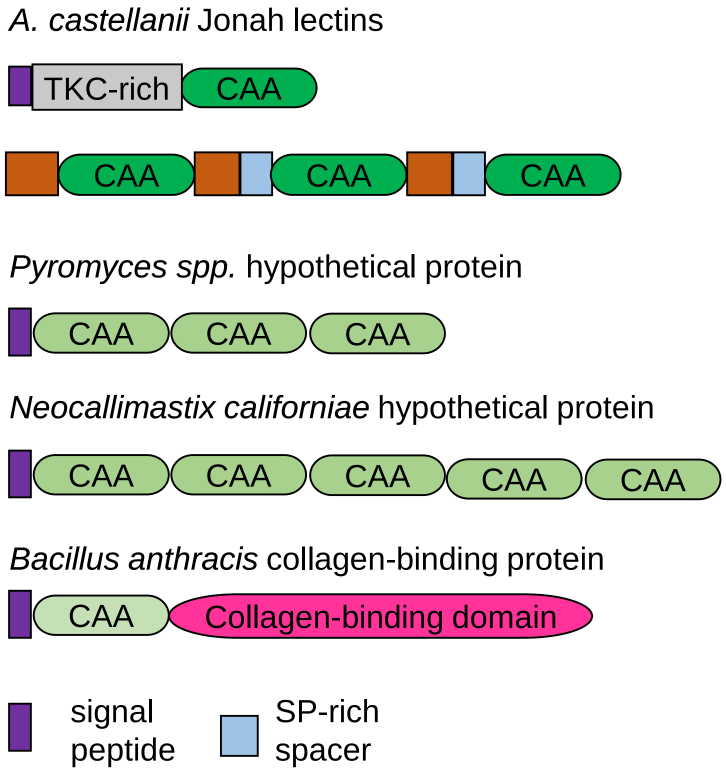
A. castellanii Jonah lectins
TKC-rich
CAA
CAA
CAA
CAA
Pyromyces spp. hypothetical protein
CAA
CAA
CAA
Neocallimastix californiae hypothetical protein
CAA
CAA
CAA
CAA
CAA
Bacillus anthracis collagen-binding protein
CAA
Collagen-binding domain
signal peptide
SP-rich spacer
